# Role of Rhodopsins as Circadian Photoreceptors in the *Drosophila melanogaster*

**DOI:** 10.3390/biology8010006

**Published:** 2019-01-10

**Authors:** Pingkalai R. Senthilan, Rudi Grebler, Nils Reinhard, Dirk Rieger, Charlotte Helfrich-Förster

**Affiliations:** Neurobiology & Genetics, Theodor-Boveri Institute, Biocenter, Julius-Maximilians University Würzburg, Am Hubland, 97074 Würzburg, Germany; pingkalai.senthilan@uni-wuerzburg.de (P.R.S.); rudi.grebler@t-online.de (R.G.); nils.reinhard@stud-mail.uni-wuerzburg.de (N.R.); dirk.rieger@biozentrum.uni-wuerzburg.de (D.R.)

**Keywords:** Rhodopsins, electroretinogram, immunocytochemistry, entrainment, Rhodopsin 7, retina

## Abstract

Light profoundly affects the circadian clock and the activity levels of animals. Along with the systematic changes in intensity and spectral composition, over the 24-h day, light shows considerable irregular fluctuations (noise). Using light as the Zeitgeber for the circadian clock is, therefore, a complex task and this might explain why animals utilize multiple photoreceptors to entrain their circadian clock. The fruit fly *Drosophila melanogaster* possesses light-sensitive Cryptochrome and seven Rhodopsins that all contribute to light detection. We review the role of Rhodopsins in circadian entrainment, and of direct light-effects on the activity, with a special emphasis on the newly discovered Rhodopsin 7 (Rh7). We present evidence that Rhodopsin 6 in receptor cells 8 of the compound eyes, as well as in the extra retinal Hofbauer-Buchner eyelets, plays a major role in entraining the fly’s circadian clock with an appropriate phase-to-light–dark cycles. We discuss recent contradictory findings regarding Rhodopsin 7 and report original data that support its role in the compound eyes and in the brain. While Rhodopsin 7 in the brain appears to have a minor role in entrainment, in the compound eyes it seems crucial for fine-tuning light sensitivity to prevent overshooting responses to bright light.

## 1. Introduction

A circadian clock has no survival value unless biological time is adjusted (entrained) to local time. To most organisms, the profound changes in the light environment provide a local time cue (called Zeitgeber). Over the 24-h day, the amount of light, its spectral composition and its direction, change in a systematic way. In theory, all these features could be used for entrainment, but each would be subject to considerable variation. This may be the reason why animals use multiple photoreceptors to entrain their circadian clocks [1,2]. Mammals, for example use the Rhodopsins in the rods and cones of their eyes and a specialized opsin, melanopsin, in the retinal ganglion cells to entrain their daily activity rhythms to the daily light–dark cycles [3,4,5,6,7]. Only the elimination of all of these photoreceptors leads to the activity rhythms that are desynchronized to environmental light–dark cycles [8]. A further opsin, Opn5, is expressed in a subset of retinal ganglion cells and was recently shown to contribute to the entrainment of the circadian clock to UV light (380 nm) [9].

Likewise, fruit flies utilize multiple photoreceptors to entrain their circadian clocks [10,11]. In addition to the six Rhodopsins in their photoreceptive organs (compound eyes, ocelli, and extra retinal Hofbauer-Buchner (HB)-eyelets) they possess the flavin-based Cryptochrome (CRY) as a cellular photopigment in the circadian clock neurons [12,13,14,15]. As light is able to penetrate the fly cuticle, the clock can directly be entrained by CRY, even in the absence of all eye structures [10]; only when CRY is additionally eliminated from all eye structures, are they called “circadian blind”, meaning that their activity rhythms cannot be entrained to the light–dark cycles [16]. Nevertheless, such “circadian blind” flies still respond to light, e.g., their activity is repressed by lights-on and stimulated by lights-off (Figure 2 in [16]), indicating that additional photopigment(s) influence their activity. The search for additional photopigments led to the detection of a seventh Rhodopsin, Rh7, that appears to participate in light-entrainment, although there are diverging results and hypotheses, concerning this finding [17,18,19,20]. 

In the present article, we will first review the effects of light on the activity rhythms of *Drosophila melanogaster* and the roles of the six conventional Rhodopsins. We will then discuss the importance of the Rh7 and add original results, showing that Rh7 is present in certain receptor cells of the compound eyes and affects their light sensitivity, as revealed by the electroretinogram (ERG) recordings. Consequently, our article presents a mixture of reviewed and original data. We show that most Rhodopsins in the eyes contribute to the entrainment of the circadian clock, as well as to the direct effects of light on the activity levels. The contribution of Rh7 to the entrainment process, appears to be relatively minor—it seems crucial for fine-tuning the light sensitivity of the compound eyes and might prevent overshooting responses to changes in light intensity.

## 2. The Role of the Eyes in Circadian Entrainment and in the Direct Light Effects on Activity

Light affects behavior in different ways. As mentioned above, cyclic changes in light intensity can act as an entraining signal, synchronizing the circadian clock, so that an animal is appropriately adjusted to the 24-h environmental period. Light can also directly modulate an animal’s activity—in diurnal animals, light often promotes activity, whereas, it inhibits activity in nocturnal animals. When such direct effects of light are present, they often conceal the activity controlled by the circadian clock, a phenomenon known as “masking” [21]. Since direct effects of light are independent of the circadian clock, they are also present in animals without a functional circadian clock, for example, in clock mutants [22] or in animals whose master clock has been surgically removed [23]. Clock-less animals can remain synchronized with the environment, in the absence of the circadian timing system, but lose rhythmicity as soon as external light–dark cycles are turned off.

Typical direct light effects in fruit flies exposed to rectangular light–dark cycles are the sudden increases in activity after lights-on in the morning and lights-off in the evening. These direct light effects last for, approximately, 30 min and are referred to as startle responses [24]. Startle responses are mediated by the compound eyes and, hence, cannot be detected in eyeless flies [10]. Under natural light–dark cycles, fruit flies do not display lights-on and lights-off responses; depending on the environmental temperature, they show activity in the morning, in the evening, and sometimes in the afternoon [25,26]. Under laboratory conditions of simulated dawn and dusk (gradual increase/decrease of light intensity within 1.5 h in the morning/evening and constant temperature), the flies shift most of their daily activity into dawn and dusk [27]. Again, this behavior depends on functional compound eyes [28]. The presence of the compound eyes is even sufficient to provoke quasi-wild-type behavior in clock-less flies, when the daily increase and decrease in light-intensity is simulated in a natural manner [29]. Thus, normal vision can partly compensate for the loss of the circadian clock under natural-like light–dark cycles. Light also influences the fly’s general activity level. Darkness and bright light inhibit activity, whereas, low light appears to promote activity [27]. When the night is illuminated by artificial moonlight, flies show considerable nocturnal activity [30]. This effect, mediated by the compound eyes, is partly due to the direct light effects that circumvent the circadian clock; the fact that clock-less flies show a similar behavior supports this evidence [31].

Nevertheless, not all light effects that are mediated by the compound eyes are direct. Moonlight, for instance, not only elevates nocturnal activity but additionally shifts the circadian clock [30]. Furthermore, the compound eyes contribute to entrainment and re-entrainment of the clock to the phase-shifted light–dark cycles (in so-called jet-lag experiments). Jet-lag experiments are widely used to test the contribution of photoreceptors in phase-shifting the clock, because they allow to distinguish between the direct light effect and real entrainment of the clock. Depending on the light-intensity, wild-type fruit flies re-entrain to the light–dark cycles that are advanced or delayed by 8 h, within one to three days [16,32]. Photoreceptor mutants need distinctively longer (up to 8 days) to do so. In the case of phase advances, CRY and the eyes were shown to equally contribute to re-entrainment, whereas in the case of phase delays, eye structures are less efficient than CRY in re-entraining the flies [32]. Nevertheless, the eyes are essential for proper phasing of evening activity, showing that they contribute to phase delays in a different ways [32]. Similarly, the eyes are essential for a proper timing of the evening activity, under long summer days [10,33]. Only flies with functional eye structures can delay their evening activity to the afternoon and evening, under such conditions. This delaying effect prevents flies from being active at midday, when the risk of desiccation is at its highest, and can consequently be regarded as an important seasonal adaptation.

## 3. Input Pathways from the Eyes to the Clock Neurons

**Figure 1:**
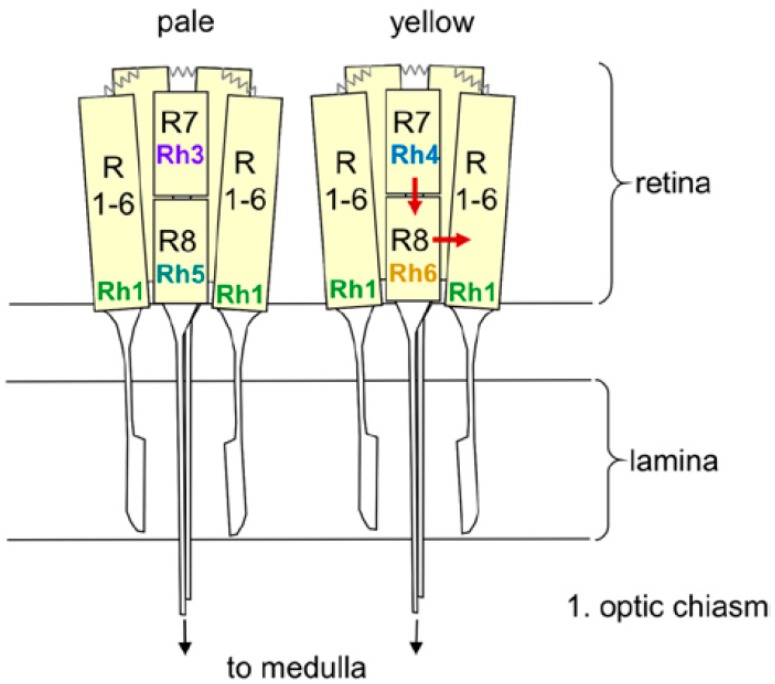
Schematic drawing of two ommatidia (pale and yellow) with detailed view of the eight receptor cells and their Rhodopsin expression. The outer receptor cells 1 to 6 (R1–6) span the entire depth of the retina, whereas the inner receptor cells R7 and R8 are arranged on top of each other. R1–6 terminate in the fly’s first optic neuropil, the lamina, whereas R7 and R8 run toward the medulla. R1–6 are connected via gap junctions (zigzag lines). The arrows indicate the putative interaction between the Rh6-expressing R8 cell and R1–6, as well as between R7 and R8 (from [40]).

**Figure 2:**
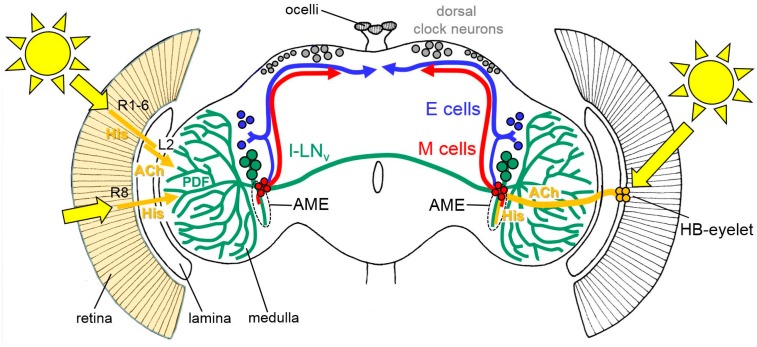
Rough schematic representation of light input through the compound eyes (left) and the Hofbauer-Buchner (HB)-eyelets (right) to the circadian lateral clock neurons (the M cells, E cells, and the large ventrolateral neurons (l-LN_v_)). All receptor cells of the compound eyes use histamine (His) as a neurotransmitter, whereas the HB-eyelets utilize histamine and acetylcholine (ACh). The HB-eyelets project into the accessory medulla (AME); they signal via histamine to the l-LN_v_, which in turn contain the neuropeptide PDF (Pigment-dispersing Factor) and via ACh to the M cells. Receptor cells 1 to 6 (R1-6) signal via the His to the lamina monopolar cells (L2). L2 cells express ACh and signal in the distal medulla to the l-LN_v_. Rh6-positive receptor cells 8 (R8) may play an integrative role in the light input from all other receptor cells but it is still unknown how they signal to the circadian clock neurons, since they are not directly connected to the l-LN_v_ [53]. The other receptor cells are omitted for clarity. For details, see text.

Each compound eye consists of ~800 ommatidia, each of which contains eight receptor cells, six outer and two inner ones (Figure 1). The outer six-receptor cells project into the lamina, where they are connected to the lamina monopolar neurons that run into the medulla (Figure 2). The inner receptor cells project directly into the medulla. Interestingly, there is considerable crosstalk between the single receptor cells [34]. The outer receptor cells are connected via gap junctions and appear to signal to the inner ones, whereas the inner receptor cells 7 and 8 have been recently shown to inhibit each other to permit color opponency [35].

Compared to that of the compound eyes, the structure of the HB-eyelets is much simpler. Each eyelet consists of four receptor cells, located at the posterior edge of each compound eye, and projects along the anterior surface of the medulla, directly into the accessory medulla—the follower of the larval optic neuropil (Figure 2) [36,37,38]. The accessory medulla serves as a hub for the light input to the circadian clock ([39], see also below).

The somata of the clock neurons are located in the lateral and dorsal brain and their neurites are extensively connected with each other (Figure 2, [41]). Several groups of clock neurons can be distinguished, some of which control the morning activity and the others control that of the evening. For simplicity, we will refer to them as Morning (M) and Evening (E) cells (for a more detailed view, see [11,41,42]). The M cells and E cells have dendrites in the accessory medulla, where they receive activating input from the HB-eyelets (Figure 2, [11,39,41]).

An additional subset of lateral clock neurons, which neither belongs to the M nor to the E cells, mediates light-dependent arousal and wakefulness [43,44,45,46]. This group is made up of the four large ventrolateral neurons (l-LN_v_). The l-LN_v_ express the neuropeptide Pigment-Dispersing Factor (PDF) and at least three of them have wide varicose arborizations in the distal medulla that are close to the terminals of the inner receptor cells and to the lamina monopolar cells downstream of the outer receptor cells (Figure 2, [41]). The l-LN_v_ also have pronounced dendrites in the ipsilateral accessory medulla and varicose terminals in the contralateral medulla, to which they project via a commissure. Similarly to the M and E cells, the l-LN_v_ receive direct input from the HB-eyelets in the accessory medulla. Furthermore, they get input from the L2 lamina monopolar cells in the distal medulla (Figure 2, [47]). The input from the L2 cells is cholinergic and activating [47], whereas, the input from the HB-eyelets is most likely histaminergic and inhibiting [48]. The two principal HB-eyelet neurotransmitters, acetylcholine, and histamine, exert opposing effects on the M cells and the l-LN_v_. Light coming to the HB-eyelets, in the morning, activates the M cells via acetylcholine, and incites the morning activity [48]. At the same time, it inhibits the l-LN_v_ via histamine. The latter inhibition appears to prevent the l-LN_v_ from firing in the morning. The l-LN_v_ reach maximal Ca^2+^ levels around the middle of the day [32,49], a time at which the cholinergic input from the compound eyes (via L2 cells) [47] might prevail. The firing l-LN_v_ release PDF that signals to the E cells and delays evening activity. This effect is most evident under long days [50] and can explain why the compound eyes are necessary in delaying evening activity, under these conditions. The activation of the M cells by the HB-eyelets may also explain why the eyes play a larger role in re-entraining fly activity to the phase-advances, compared to the phase-delays ([32] and above). Similarly to light, an artificial temporary activation of the M cells by TRP (transient-receptor-potential) -channels, in the subjective morning, incites the prominent phase-advances. However, contrary to light, the same temporal activation of the M cells in the subjective evening cannot provoke phase-delays [51], indicating that phase-delays are not accomplished by the M cells. Most likely, phase delays are mediated by an activation of CRY in the E cells [52].

## 4. Roles of Rhodopsins 1 to 6 in Entrainment and Direct Light Effects

Our description of the light input signal from the compound eyes and the HB-eyelets has so far considered these structures as a whole, however, the following section will instead focus on the five different Rhodopsins of the compound eyes and their different effects on fly activity. The six outer receptor cells express Rhodopsin 1 (Rh1), which has a broad sensitivity to blue-green light, whereas the inner receptor cells can be divided into two subtypes (Figure 1). In the “pale” cluster, receptor cell 7 expresses the ultraviolet (UV)-sensitive Rhodopsin 3 (Rh3) and receptor cell 8 shows blue-sensitive Rhodopsin 5 (Rh5). In the “yellow” cluster, receptor cell 7 contains Rhodopsin 4 (Rh4), which is sensitive to longer UV wavelengths and receptor cell 8 shows the green sensitive Rhodopsin 6 (Rh6). The pale and yellow subtypes are stochastically distributed in a ~30:70 ratio throughout most of the retina, whereby the Rhodopsin expression is regulated by sophisticated molecular mechanisms [54]. The four HB-eyelet cells express Rh6, as does the receptor cell 8 in the “yellow” ommatidia. The ocelli, instead, express Rhodopsin 2 (Rh2), which is neither present in the compound eyes nor in the HB-eyelets.

The contribution of the different receptor cells to the entrainment, as well as to direct effects of light, was tested by recording the different combinations of Rhodopsin mutants. Saint-Charles et al. [32] tested the re-entrainment of the Rhodopsin mutants to 8 h phase-advances and -delays of the light–dark cycles and found that four of the six Rhodopsins can mediate the re-entrainment—Rh1, Rh3, Rh4, and Rh6. No re-entrainment was found when all Rhodopsins, except Rh2, were eliminated, suggesting that the ocelli alone are not able to entrain the clock. Similarly, the Rh5-positive receptor cells 8, alone, were not able to entrain the flies.

Schlichting et al. [40] tested the direct light effects of the different Rhodopsins on activity, by determining fly nocturnal activity levels under moonlit nights and highly illuminated days. The authors found that the Rh6-expressing inner receptor cells 8, together with the Rh1-expressing outer receptor cells, mediate the increase in the relative nocturnal activity, in response to moonlight, whereas, only Rh1 of the outer cells seems necessary for nocturnal activity to increase in response to raising daylight intensities. The Rh6-expressing HB-eyelets played no role in moonlight detection but contributed to the proper phasing of evening activity. A prominent influence of the HB-eyelets to the phasing of the evening activity and consequently to the length of the siesta was shown at high light intensities (~8000 lux), by a very recent study [55]. Furthermore, Alejevski et al. [53] demonstrated a prominent role of the Rh6-positive receptor cells 8 in entrainment—all histaminergic inputs from the outer and the inner receptor cells appear to converge to these Rh6 cells, in order to contribute to the circadian entrainment. This is reminiscent of the circuit logic of circadian entrainment in the mammalian retina, where intrinsically photoreceptive retinal ganglion cells receive inputs from the rods and cones and additionally express melanopsin [6,56]. The mammalian circadian clock can entrain to the day–night cycles by tracking the color changes, in addition to the intensity changes [57]. Future studies are yet to determine if such properties are encoded by the Rh6-expressing receptor cells in the fly retina. With exception of two studies using green, yellow, or red light, during the light phase of the light–dark cycle [58,59], no systematic recordings under the colored light have been carried out in the *Drosophila*. The authors found that the Rh1-expressing photoreceptors 1–6 and the Rh6 expressing photoreceptors 8 are required for the entrainment to red light, and that entrainment to green and yellow light was abolished in *cry, Rh1, Rh5,* and *Rh6* quadruple mutants. These results fit the spectral sensitivities of the relevant Rhodopsins [60] and underline the contribution of multiple photoreceptors to entrainment.

In summary, we can conclude that all photoreceptors of the compound eyes and the HB-eyelets contribute to entrainment, while the Rh6-positive receptor cells appear to play a prominent role in this process. The Rh6-positive receptor cells 8 of the compound eyes may even work as integrators of light signals coming from the other receptor cells in the compound eye [53]. Nevertheless, the downstream interneurons of the receptor cells 8 that signal to the circadian clock neurons remain unknown. Furthermore, it is, yet, unknown how these cells integrate the signals deriving from the other receptor cells. In this context, two interesting recent studies suggest that the Rh6-positive receptor cells 8 contain a second Rhodopsin (Rh7) that does not activate the phototransduction cascade but might interfere with intercellular signaling of the Rh6 [18,19]. These studies are in apparent contrast to a third study reporting that Rh7 acts as a deep brain photoreceptor directly in the l-LN_v_ [20]. As exemplified above, the l-LN_v_ are themselves important light-input and arousal neurons, on which the light signaling from the compound eyes and the HB-eyelets converge. They might, therefore, be similar light input integrators to that of the Rh6-positive receptor cells 8, and the putative presence of Rh7 in them, is very exciting.

In the following, we will first review the contradicting results on Rh7 and then add new preliminary results supporting the hypothesis that Rh7 plays a role in the compound eyes.

## 5. Diverging Hypotheses Concerning the Role of Rh7

Compared to the other Rhodopsins, Rh7 is probably the most unconventional one. Rh7 was first described in the year 2000, when the whole genome of the fruit fly *Drosophila melanogaster* was sequenced [61]. Rh7 possesses nearly all important features of a functional Rhodopsin, but differs from other Rhodopsins in its genomic, structural, and expressional properties, suggesting its unique role [17]. Examples are the untypical RCSI (Rhodopsin Core Sequence I) motif and the lack of the QAKK domain. RCSI is a cis-regulatory element found ca. 100 bp upstream of the transcription starting site that is conserved in all *Drosophila* genes coding for the phototransduction proteins [62]. While this sequence is palindromic, in general, phototransduction genes that are broadly expressed in all receptor cells, is non-palindromic in the genes coding for Rhodopsins, indicating its role in the cell-type specific expression. In Rh7, this sequence is palindromic and found in the middle of the gene, in the second intron. Rh7, furthermore, lacks the QAKK domain, which is highly conserved in the insect Rhodopsins and which is supposed to activate the G-protein coupled signaling pathway directly. Only Rh7 and its homologues in other insects seem to lack this domain [17].

### 5.1. Testing the Function of Rh7 as a Photopigment

To test whether Rh7 is a functional photopigment, Grebler et al. [18] expressed Rh7 under the control of the *Rh1* promotor in *Rh1*-lacking flies (*ninaE^17^* mutants) and tested whether Rh7 can assume Rh1’s role. They detected an up-to-10,000-fold enhanced *Rh7*-RNA expression, which is equal to the RNA expression of *Rh1*, indicating that the expression worked. *ninaE^17^* mutants undergo a typical eye degeneration process due to the loss of Rh1, which is known to be highly enriched in the cell membrane, where it is thought to stabilize the outer receptor cells. Grebler et al. [18] observed a delayed eye degeneration in *ninaE^17^* mutants expressing Rh7, indicating that Rh7 is indeed assembled in the cell membrane of the outer receptor cells and partly overtakes the function of Rh1. However, Rh7 was not able to replace the light signaling abilities of Rh1, since it could not rescue the wild-type electroretinogram (ERG) responses in the *ninaE^17^* mutants [18]. Grebler et al. [18] concluded that this effect is due to the lack of the QAKK domain or due to a possible, different Rh7-signaling cascade, which is not present in the outer receptor cells [17].

Nevertheless, Ni et al. [20] came to different conclusions. Using a slightly different method, they were able to rescue the ERGs in *ninaE^17^* mutants by expressing Rh7. Their *ninaE^17^* mutants carried an additional mutation in the *norpA* gene coding for Phospholipase C (PLCß), which is a central protein in the phototransduction cascade. Flies lacking NorpA had no ERGs, while flies lacking only Rh1 still showed ERG responses of the inner receptor cells. Ni et al. [20] then rescued *norpA* and simultaneously expressed *Rh7* in the outer receptor cells. By doing this, they eliminated the responses of the inner receptor cells and only monitored the responses of Rh7 in the outer receptor cells. This method is regarded as more sensitive than the method employed by Grebler et al. [18] and, indeed, Ni et al. [20] could see the ERG responses in the rescued flies. Ni et al. [20] used slightly different light parameters, they did not check the photoreceptor degeneration in their *ninaA norpA* double mutants rescued flies, and did not provide ERG dose-response curves for the control and the rescued flies; nevertheless, the differing results of Ni et al. [20] and Grebler et al. [18] are hard to understand.

### 5.2. Place of the Rh7 Action

The expression level of the *Rh7* was found to be very low in all three studies. Kistenpfennig et al. [19] found equally low *Rh7* levels in isolated brains and retinas, whereas Ni et al. [20] concluded that Rh7 is not expressed in the retina as they found similar *Rh7* RNA levels in wild-type flies and *glass^60j^* mutants, in which ocular receptor cells are absent.

Kistenpfennig et al. [19] could not see any labeling with Rh7 antibodies in the wild-type flies, but after overexpressing Rh7 in the outer receptor cells, the antibodies gave significant signals, which proved their specificity. The authors concluded that the expression level of Rh7 is below the detection limit of their antibodies. To localize Rh7 in the brain and retina, they tested a *Mi{Mic}* fly line that carries the *gfp* reporter gene, within the first intron of the *Rh7*. GFP labeling was found in the fenestrated layer located between the retina and the lamina, in the HB-eyelets, and in the *Rh6*-expressing inner receptor cells 8 of the retina. In addition, GFP staining was present in many brain and optic lobe cells, including some dorsal clock neurons that have previously been implicated in the control of the siesta. No staining was found in the lateral M and E clock neurons [19]. In contrast, Ni et al. [20] reported Rh7 immunostainings in the l-LN_v_ and the M cells and, even more excitingly, they found that the l-LN_v_ responded to UV light (400nm), with an increase in firing rate that was significantly lower in an *Rh7^1^* mutant (see below). Rh7 signaling in the l-LN_v_ activates a transduction cascade different to that of the compound eyes, which involves the PLCß encoded by the *norpA* gene. In the l-LN_v_, the PLC21C has overtaken the role of PLCß [20].

Again, the differing results of Kistenpfennig et al. [19] and Ni et al. [20] are hard to reconcile.

### 5.3. Effects of the Rh7 on the Entrainment of the Circadian Clock

**Figure 3:**
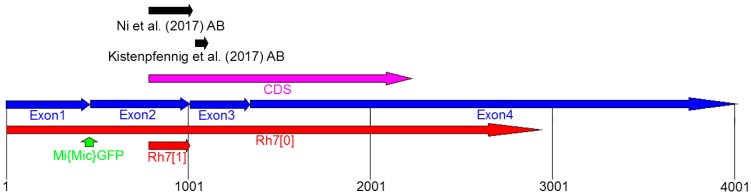
Genomic organization of the *Rh7* locus. *Rh7* possesses four exons (blue) and the code sequence (CDS) is located between Exon2 and Exon4 (magenta). Both, the *Rh7* mutants (*Rh7^0^* [19] and *Rh7^1^* [20]) have different deletions in the *Rh7* gene (shown in red). The *gfp* insertion site of the *Mi{Mic}*-line is located between Exon1 and Exon2 (green arrow). The antibodies used by Ni et al. (2017) and Kistenpfennig et al. (2017) are raised against the different parts of the Rh7 (indicated by black arrows).

Kistenpfennig et al. [19] and Ni et al. [20] generated *Rh7* mutants and *Rh7 cry^0^* double mutants, in order to test the role of Rh7 in the circadian entrainment. *Rh7^0^* mutants were created by p-element-mediated mutagenesis and carried an, approximately, 10.35-kb deletion that extended over the entire *Rh7* coding sequence (Figure 3) [19]. *Rh7^1^* mutants were created by an ends-out homologous recombination, which led to deletion of 540 base pairs of the *Rh7* gene, including the translation start site and the first 80 amino acids of the Rh7 [20] (Figure 3).

As can be expected from the previous chapters, the claimed effects of the Rh7 mutants on rhythmic activity were quite diverging between the studies. While Kistenpfennig et al. [19] only reported minor roles of the Rh7 in circadian entrainment, Ni et al. [20] reported profound impairment in photo entrainment, at least in their *Rh7 cry^0^* double mutants. 

However, at a closer inspection the results appear less diverging. Kistenpfennig et al. [19] found their *Rh7^0^* mutants and *Rh7 cry^0^* double mutants to exhibit less morning activity and a longer siesta than the wild-type controls. Ni et al. [20] did not analyze the activity pattern of their flies in detail, but a similar phenotype might be present in their actograms, especially in the double mutants. Ni et al. [20] found that their *Rh7^1^* mutants take slightly longer to re-entrain to a 6-h phase-delay of a white light–dark cycle, while Kistenpfennig et al. [19] found similar differences for their *Rh7^0^* mutants under blue light (470nm), but not under white light. Under constant white light, which is known to make the wild-type flies arrhythmic, Ni et al. [20] found that more *Rh7^1^* mutants remained rhythmic, as compared to the controls, at least in low-light intensities (10 lux). At higher light intensities (400 lux), the differences disappeared. The reason why Kistenpfennig et al. [19] did not see differences between their *Rh7^0^* mutants and the control flies might be due to the fact that they only tested 1000 lux, an intensity which appeared to be much too high to reveal the effect seen by Ni et al. (2017). Furthermore, Ni et al. [20] found that the *Rh7^1^* mutants barely responded to short light-pulses and showed lower arousal during 5 min light pulses, at late night. These two assays were not tested by Kistenpfennig et al. [19].

In summary, both studies found effects of the Rh7 on the circadian entrainment and direct effects of light, which speaks for a role of Rh7 in circadian photoreception. The different experimental protocols, as well as the use of *Rh7* mutants with different genetic backgrounds, may explain the dissimilarities between the two studies.

On the whole, clear discrepancies existed in the proposed location and manner of action of the Rh7. Does the Rh7 work as a deep brain circadian photoreceptor in the l-LN_v_ cells or does it have a role in the Rh6-expressing photoreceptor cell 8? Can Rh7activate the phototransduction cascade in the photoreceptor cells, by itself, or does it work via other Rhodopsins?

## 6. New Results Reveal a Role of the Rh7 in Compound Eyes

To clarify the diverging results concerning the location and action of the Rh7, we have carried out further experiments. We have performed additional quantitative PCR (qPCR) analysis, improved our immunostainings with an Rh7 antibody and recorded ERGs of the wild-type flies and the *Rh7^0^* mutants to elucidate a putative role of the Rh7 in light-sensitivity of the compound eyes.

### 6.1. Materials and Methods

● qPCR

The relative mRNA levels of all Rhodopsins in the brain and in the retina, were quantified via qPCR. Total RNA extraction, reverse transcription, and qPCR were carried out as described by Grebler et al. [18]. For each strain and tissue, three biological replicates were examined (brains or retinas of five flies per replicate), and, for each replicate, two PCRs were run. The relative mRNA levels were calculated using the ΔCT equation and *alpha-tubulin* was used as the reference gene. Primer sequences are shown in Table 1.

● Generating a UAS-Rh7 construct

Full-length *Rh7* cDNA was amplified by PCR from pOT2 vector of a commercially available cDNA clone, GH14208, using a primer pair creating restriction enzyme sites. After restriction enzyme digestion and amplification, the purified PCR product (1.8 kb) was ligated into the pUAST expression vector using the EcoRI restriction sites. The cDNA insert was confirmed by sequencing after the “in sense” direction by digestion with XhoI. In addition, we tested the Rh7 RNA expression in each fly line, via qPCR.

● Immunohistochemistry

Preparation and immunostainings of retinas were performed, as described by Kistenpfennig et al. [19] and preparation and immunostainings of the whole-mount brains were performed, as described by Hermann et al. (2012). Primary antibodies were applied in 0.5% PBT, 0.02% Sodium azide, and 0.4% NGS, for 2 days, at 4 °C. Anti-Rh7 antibody was pre-incubated in the *Rh7^0^* embryos, prior to incubation. The following antibody concentrations were used: Rabbit anti-Rh7 (peptide antibody against aa86-110 [19]) at 1:100, mouse anti-Rh1 (Developmental Studies Hybridoma Bank, University of Iowa) at 1:100, and the mouse anti-pigment-dispersing factor (PDF) (Developmental Studies Hybridoma Bank, University of Iowa) at 1:1000. In brains and retinas, fluorescent secondary antibodies (Alexa Fluor 488, 647; Invitrogen, Carlsbad, CA, USA) were applied for 9 h at room temperature. Immunostainings were visualized by confocal microscopy (TCS SP8; Leica, Wetzlar, Germany). Staining intensity in the cytoplasm of the l-LN_v_ cells was determined on single confocal stacks, as described in [27]. 

● Electroretinogram (ERG)

ERG recordings were performed, as described in Grebler et al. [18]. If not otherwise stated, flies were dark-adapted for 15 min, before the experiment. Either an LED-based illumination system (pE-4000; CoolLED Ltd., Andover, UK) was used to generate the UV light stimuli (365 nm) or a halogen lamp was used (Spindler & Hoyer, Göttingen, Germany) to generate white light stimuli of 400 ms duration and different intensities. The receptor-potential amplitudes of the electroretinogram (ERG) responses were plotted as a function of the relative light intensity (in logarithmic units), to yield the irradiance response curves. Each curve was obtained from n = 7–13 flies. The sum of the measured receptor potential amplitudes for all light intensities (or separately for low and high light intensities) were calculated for each single fly and statistically compared between the different strains. In all experiments, the recorded flies had the same eye-color (red or white), to eliminate any influence of eye pigmentation.

### 6.2. Results

#### 6.2.1. Rh7 is Expressed in the Compound Eyes and in the Brain, but Not in the l-LN_v_

**Figure 4:**
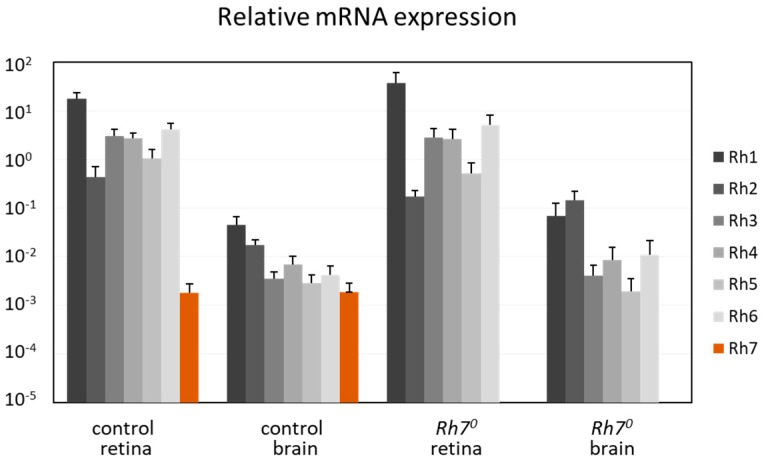
qPCR: Relative mRNA expression of all Rhodopsins in comparison to α-tubulin, in the brains and in retina of the control and the *Rh7^0^* mutant flies. All Rhodopsins were expressed in the brain and in the retina. Rh1 to Rh6 were highly expressed in the retina, but were about 100 times weaker in the brain. Rh7 was weakly, but equivalently expressed in the brain and in the retina.

We determined the mRNA levels of all Rhodopsins in the brain and in the retina, by comparing with α-tubulin (Figure 4). RNAs from all Rhodopsins (*Rh1* to *Rh7*) seemed to be expressed in the brain and in the retina. While *Rh1* to *Rh6* were highly expressed in the retina, they were about 100–1000 times weakly expressed in the brain. In contrast, the *Rh7* RNA was weakly, but equivalently, expressed in the brain and in the retina and absent in the *Rh7^0^* mutants. This weak expression of the *Rh7* was equal to the weak expression of the *Rh1* to *Rh6* in the brain. 

**Figure 5:**
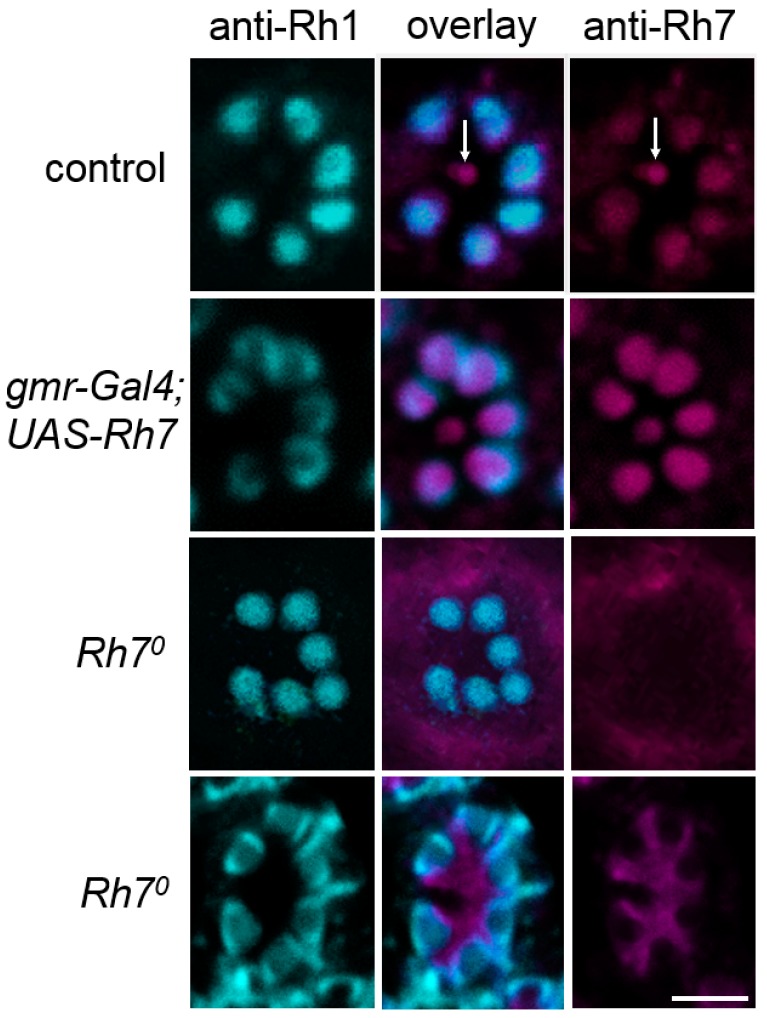
Single confocal stacks (2µm thick) of whole-mount retinas labeled with anti-Rh1 (cyan) and anti-Rh7 (magenta). Labeling is shown for control flies, flies with Rh7 overexpressed in all photoreceptor cells (*gmr-Gal4;UAS-Rh7*), and the *Rh7^0^* mutant flies. While the anti-Rh1 antibody stained the rhabdomeres of the six outer receptor cells, the anti-Rh7 weakly stained those receptor cells, particularly in that of the inner receptor cell R8 in the control flies (arrows). In *gmr-Gal4;UAS-Rh7* flies, Rh7 staining was increased in all rhabdomeres. *Rh7^0^* mutants showed no staining in the rhabdomeres. Either a low background staining was found (upper *Rh7^0^* row) or the inter-rhabdomeric space was stained (lower *Rh7^0^* row), as found previously [18]. Please note that Rh1-labeling was sometimes only present at the margin of the outer photoreceptor cells (here seen in *gmr-Gal4;UAS-Rh7* flies and in the lower row of *Rh7^0^* mutants). Scale bar: 5µm.

Furthermore, we improved the immunostaining with our peptide antibody, which was raised against the N-terminal amino acids 86–110 of the Rh7 [19]. Consistent with our previous results, using the *Mi{Mic}*-line, we observed weak Rh7 staining in all receptor cells in the retina, a staining that was most intense in the inner receptor cells 8. We could not see any staining in the retina of *Rh7^0^* mutants and a stronger staining was seen in the outer receptor cells of the flies that were overexpressing Rh7 in all photoreceptor cells (Figure 5).

**Figure 6:**
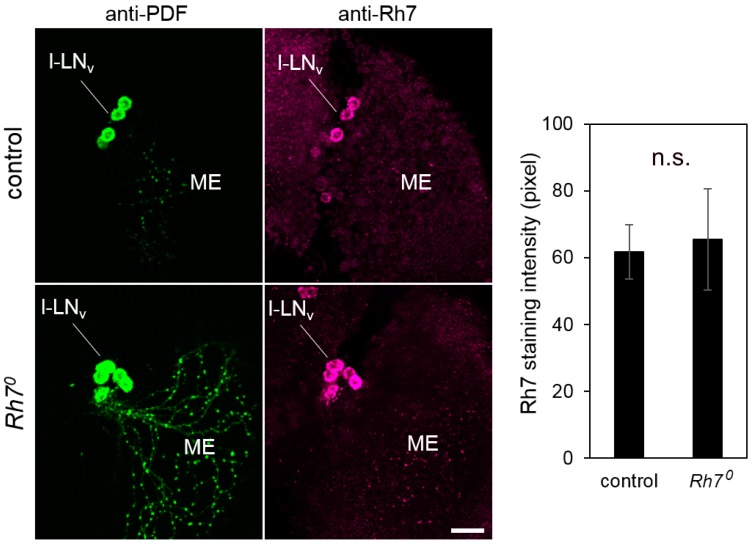
Co-immunostainings with anti-Rh7 and anti-PDF antibodies in the l-LN_v_ cells of the control flies and the *Rh7^0^* mutants (overlay of five confocal stacks, each). We observed staining of the same intensity in the l-LN_v_ cells of the control flies and the *Rh7^0^* mutants. The staining was quantified in twelve brain hemispheres of the controls and the mutants, respectively (error bars indicate standard errors of the means). Scale bar: 20 µm.

To clarify the Rh7 expression in the brain, we co-stained the whole-mount brains of the controls and the *Rh7^0^* mutants, with anti-PDF and anti-Rh7 antibodies. We observed that the anti-Rh7 stained some dorsal neurons, as well as some PDF-positive l-LN_v_ in the control brains (Figure S1). In the *Rh7^0^* mutants, the staining in the dorsal neurons was no longer visible, but the staining in the PDF-positive l-LN_v_ was still evident (Figure S1 and Figure 6), although the Rh7^0^ mutants lacked the whole Rh7 coding region and even did not express the *Rh7* mRNA (Figure 3, Figure 4). We conclude that the Rh7 staining in the l-LN_v_ was, at least, partly unspecific. The same may apply for the antibody staining of the Ni et al. study [20]. Although both Rh7 antibodies were raised against different epitopes, we found that there were at least two other *Drosophila* proteins that could be detected with both antibodies: Msp300 and CG6424 (Table S1). The antibody used in Ni et al. [20] was raised against the GST-fused Rh7-N-terminus, but we only considered the Rh7-part of this epitope in this analysis. Considering the entire GST-Rh7 epitope may even lead to more unspecific binding sites.

Our results strongly suggest that the prominent Rh7 labeling in the l-LN_v_ cells is unspecific. The RNA_seq_ experiments corroborate our results that *Rh7* is not preferentially expressed in the l-LN_v_ [63]: *Rh7* was not among the genes with the highest expression in the circadian clock neurons, as compared to the non-clock neurons. However, the patch-clamp recordings of Ni et al. [20] showed that the firing frequencies of the l-LN_v_ neurons, in response to blue light (405 nm), were diminished in the *Rh7^1^* mutants, indicating that Rh7 was working in the l-LN_v_ cells. Our qPCR results showed that *Rh7* mRNA is expressed, in equal levels, in the brain and the retina. Rh7 might, therefore, be present at levels below the detection limit of the antibodies, in many brain cells, including the l-LN_v_. These very low levels may be sufficient to induce electrophysiological responses to blue light, as was described by Ni et al. [20]. 

#### 6.2.2. Rh7 Reduces the Magnitude of the ERG

**Figure 7:**
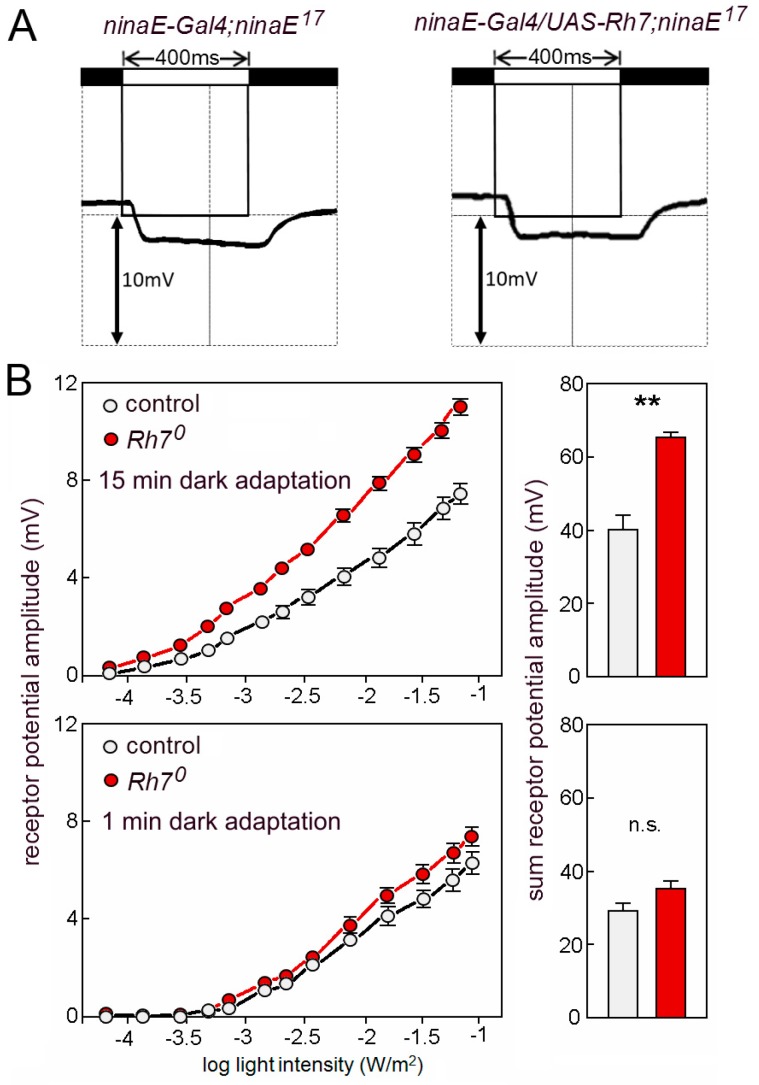
(**A**) ERGs using 400 ms light pulses of UV light (365nm). *ninaE^17^* mutant controls and *ninaE^17^* mutants that express *Rh7* in the outer receptor cells R1–R6 lack the lights-on and the lights-off transients and show the same low receptor potential that stems from the inner receptor cells. (**B**) Rh7 reduces the ERG amplitudes to white light in dark-adapted flies. Irradiance response curves of the receptor potential amplitudes in the white-eyed *Rh7^0^* mutants and the relevant white-eyed controls in dark- (top) and light-adapted (bottom) flies. The statistical comparison of the summed receptor potentials is shown in the right panels.

Grebler et al. [18] have tested the ERGs of *ninaE^17^* mutant flies that express *Rh7* instead of *Rh1*, to find out whether Rh7 can rescue the *ninaE* mutants. Due to the contradicting results with Ni et al. [20], we have repeated this experiment. This time, we used the UAS-Gal4 system [64] and we expressed the *UAS-Rh7* under the control of *ninaE-Gal4*. We found that Rh7 was still unable to rescue the reduced ERGs of the *ninaE^17^* mutants (Figure 7A). 

We continued to characterize the *Rh7^0^* mutants and compared the magnitude of the ERG responses between the mutants and the relevant controls (revertants). To our surprise, we found that the same amount of white light evoked larger receptor potentials in the *Rh7^0^* mutants than in the control flies (Figure 7B). The larger ERG amplitudes were only visible in flies that were dark-adapted for 15 min. Flies that were measured after 1 min dark adaptation did not significantly differ from controls (Figure 7B). This indicates that the Rh7-dependent reduction of the ERG amplitude relies on a physiological photoreceptor state that is acquired during the dark adaptation of the eyes.

**Figure 8:**
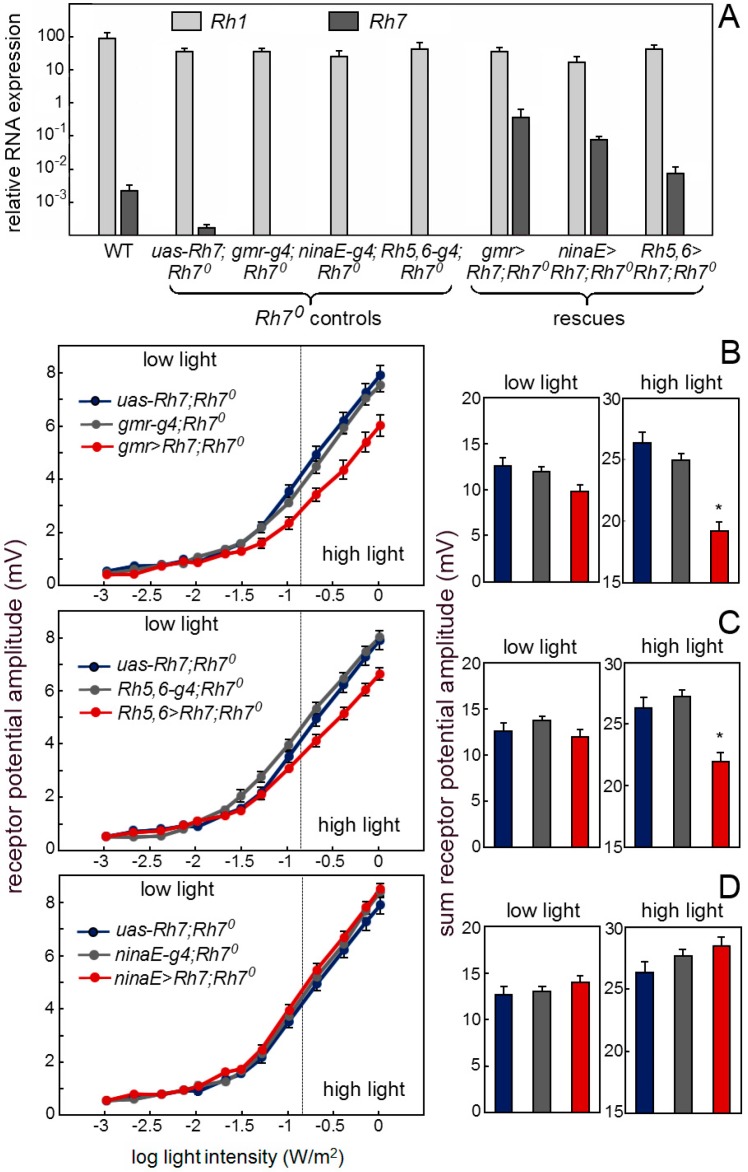
Rh7 expression in the receptor cells 8 rescues the wild-type ERG. (**A**) Rh7 expression revealed by qPCR in the retina of the wild-type controls, *Rh7^0^* mutant controls and flies, in which Rh7 was rescued in different receptor cells (*gmr*>*Rh7;Rh7^0^*: all receptor cells; *ninaE>Rh7;Rh7^0^*: the outer receptor cells *R1-6; Rh5,6>Rh7;Rh7^0^*: receptor cell R8). (**B**) Irradiance response curves of the receptor potential amplitudes in the dark-adapted flies of the *Rh7^0^* mutant controls and the *Rh7^0^* mutants, in which Rh7 was rescued in all receptor cells (*gmr>Rh7;Rh7^0^*). Under high light, the presence of Rh7 in all photoreceptors significantly reduces the ERG amplitude. (**C**) Flies with Rh7 rescued only in receptor cells 8 (*Rh5,6>Rh7;Rh7^0^*) also showed significantly lower ERG amplitudes than the *Rh7^0^* controls at high light. (**D**) The ERG responses of the flies with Rh7 expressed only in the outer receptor cells (*ninaE>Rh7;Rh7^0^*) did not differ from the *Rh7^0^* controls. All flies were in a red-eyed *y^−^ w^+^*-background to ensure equal eye color.

Our antibody staining against the Rh7 and the *Mi{Mic}*-line indicate that Rh7 is expressed in the receptors cells and particularly in receptor cells 8 of the fly retina. If this is true, we should be able to rescue a wild-type ERG in the *Rh7^0^* mutants by expressing *Rh7* in the retina. First, we expressed *Rh7* in the entire retina by using the *gmr-gal4* (*gmr-g4*) driver that expresses, strongly, in all receptor cells (Figure 8A). We found that the *Rh7* expression in all receptor cells lowered the receptor potential amplitude to the wild-type level (Figure 8B). The same was true after rescuing the *Rh7* with *Rh5-gal4*,*Rh6-gal4* (*Rh5,6-g4*), in the receptor cells 8, only (Figure 8C). However, we did not see any reduction of the ERG amplitude, when using the *ninaE-gal4* (*ninaE-g4*), which was strongly expressed, specifically in the outer receptor cells (Figure 8D). Together, these results showed that Rh7 in the retina is responsible for the observed reduction of ERG amplitude and that it acts predominantly in receptor cells 8. Most interestingly, Rh7 reduced the ERG amplitude only at higher irradiances and this fits nicely to its presumed action in the receptor cells 8. Since the inner receptor cells are less light-sensitive than the outer ones [65], their contribution to the ERG can only be seen at higher irradiances.

### 6.3. Discussion

Our results showed that Rh7 is expressed at rather low levels in the brain and in the compound eyes of fruit flies. In the brain, we could not reproduce the results of Ni et al.’s [20] findings, as we did not detect the presence of Rh7 in the l-LN_v_ by immunocytochemistry. Nevertheless, Rh7 appeared to be present at low levels in many brain neurons, probably also in the clock neurons and this may explain its contribution to circadian entrainment, as suggested by Ni et al. [20], and to the form of the siesta, as proposed by Kistenpfennig et al. [19].

In the compound eyes, Rh7 appeared to be particularly present in the receptor cells 8, and in contrast to our expectations, it tended to reduce the light-sensitivity of the compound eyes rather than increasing it. These experiments certainly need repeating, but they fit to the observation of Grebler et al. [18], who found that Rh7 cannot activate the phototransduction cascade in the outer receptor cells and, thus, did not work as a conventional photopigment. Here, we confirmed the latter results by using the UAS-Gal4 system (Figure 7A). Rh7 might interact with Rh6 and, hence, impede signaling, as has been shown for the other G-protein-coupled receptors [66,67]. Currently it is hard to say whether this can explain the alterations in the activity pattern observed in the *Rh7^0^* mutants. Rh7 in the receptor cell 8 can neither influence entrainment to low light, nor the observed effects of low light on rhythmicity [20], because the reported ERG effects are only present at a high-light intensity in the dark adapted flies. Thus, Rh7 in the receptor cell 8 only contributes to photoreception in bright light and, preferentially, in dark-adapted flies. It is imaginable that Rh7 buffers the response of receptor cell 8 to sudden changes from dark to bright light, e.g., those which occur in the morning under rectangular light–dark cycles. This could explain the altered lights-on response and morning activity of the *Rh7^0^* mutants, as compared to the wild-type flies [19], but such rectangular light–dark cycles do not occur in nature, questioning the adaptive value of the Rh7. Nevertheless, since the Rh6-positive receptor cells 8 appear to integrate the responses of all receptor cells in the eyes [53], it is possible that the Rh7 modulates the general sensitivity of the compound eyes to bright light and perhaps prevents the flies from overstimulation, even under natural conditions. Rh7 is highly conserved in arthropods and even present in several homologues, in species exposed to bright light, such as pea aphids [17]. On the other hand, bees and ants, that are phylogenetically closely related to fruit flies, but spend a considerable amount of their life in dark hives, lack Rh7. In summary, Rh7 might improve non-image-forming vision in insects in bright light conditions. Along with contributing to the circadian entrainment, its primary role may lie in fine-tuning the light-sensitivity of the compound eyes; in particular, it may prevent overshooting responses to quickly fluctuating bright light. Melanopsin similarly modulates light-sensitivity of the mammalian eye by mediating the pupillary reflexes, besides entraining the circadian clock [8].

## 7. Conclusions

In the present article we discussed that most Rhodopsins in the eyes (compound eyes and HB-eyelets), including the novel Rh7, contribute to the non-image-forming vision, such as the entrainment of the *Drosophila*’s circadian clock and to the direct light effects on the activity level. Although superficially different, flies and mice use multiple photopigments and partly specialized photoreceptor mechanisms (employing novel photopigments). We conclude that this multiplicity of photic inputs in highly divergent organisms, must relate to the complex sensory task of using light as a Zeitgeber.

## Figures and Tables

**Table 1:** Primers used in the qPCR.

Gene	5′-Primer (5′-3′)	3′-Primer (5′-3′)
*Rh1* *Rh2* *Rh3* *Rh4* *Rh5* *Rh6* *Rh7* *alpha-tubulin*	TGCCTACATCTGGTTCATGTCGAGC GCCATCCCAAGTACCGCATAGTTCTCA TGCGAACTCCCTCCAATATACTGGTC GAACCGCAACATGACCTTCACCAAG TTCTCCGTGCTGCCATTGTTCCAG ATATCGCTGTGGTTCTTCGCCTG CATCTGCGACTTTCTGATGCTCATC TCTGCGATTCGATGGTGCCCTTAAC	GGAGTAGAAGATCAGGTATGAGCGTG CACGATTTAAGCCTTTGAATCCGCCTC TATAAACGCATTGGTGGCACCGGC CAAACAACCGGGTGTCAAAGTTGTCC GTGAAGAGCTTGTAGTAGGACACCAGG CTGCTTGTACTTCGGGTGGCTC GGATGCACCACCACATTGTACCGATC GGATCGCACTTGACCATCTGGTTGGC

## Supplementary Materials

The following are available online at http://www.mdpi.com/2079-7737/8/1/6/s1, Figure S1: PDF and Rh7 labelling in the right brain hemispheres of a control fly and an *Rh7^0^* mutant. Table S1: TOP 250 Proteins that could be detected by the peptide antibody (against aa86-110) used in Kistenpfennig et al. [19]. Table S2: TOP 250 Proteins that could be detected by the antibody (against aa1-80) used in Ni et al., [20].
