# Supplementary material for: Role of Rhodopsins as Circadian Photoreceptors in the Drosophila melanogaster
Source: Biology (Basel). 2019 Jan 10;8(1):6. doi: 10.3390/biology8010006 (PMC6466219; doi:10.3390/biology8010006)
Supplement: Supplementary file 1 [file biology-08-00006-s001.pdf]

biology MDPI

Role of Rhodopsins as Circadian Photoreceptors in the *Drosophila melanogaster*

Pingkalai R. Senthilan, Rudi Grebler, Nils Reinhard, Dirk Rieger, and Charlotte Helfrich-Förster

To find out Drosophila proteins that could be identified by Rh7 antibodies used in Ni et al. [20], and Kistenpfennig et al. [19], we used a BLASTP search (https://blast.ncbi.nlm.nih.gov/Blast.cgi) for short input sequences (expect threshold: 200,000, word size: 2, matrix: PAM30, gap costs: existence: 9 extension: 1, no adjustment, taxid: 7227). For the search, we used the epitope sequence aa86 to aa110, for the Kistenpfennig et al. [19] antibody, and the epitope sequence aa1 to aa80, for the Ni et al., [20] antibody. We restricted the search to the first 250 similar protein sequences for both epitopes and looked for common proteins in both searches. We found three proteins: Rh7, Msp300, and CG6424 that could be detected by both antibodies (indicated in red). One should consider that the antibody used in Ni et al., [20] was raised against the GST-fused Rh7 [aa1-aa80] peptide. Taking the entire GST-Rh7 epitope in account, may lead to even less-specific binding sites.

Table S1. TOP 250 Proteins that could be detected by the peptide antibody (against aa86-110) used in Kistenpfennig et al. [19]).

Description	Max Score	Total Score	Query Cover	E Value	Ident	Accession
rhodopsin 7 (Drosophila melanogaster)	91.0	105	100%	3e-23	100%	NP_524035.2
ACXC, isoform A (Drosophila melanogaster)	27.4	41.6	52%	0.51	78%	NP_609593.1
uncharacterized protein Dmel_CG13613 (Drosophila melanogaster)	26.5	26.5	40%	0.97	39%	NP_001097900.2
BIR repeat containing ubiquitin-conjugating enzyme, isoform C (Drosophila melanogaster)	26.5	67.5	76%	1.0	69%	NP_001262461.1
BIR repeat containing ubiquitin-conjugating enzyme, isoform E (Drosophila melanogaster)	26.5	67.5	76%	1.0	69%	NP_001262463.1
BIR repeat containing ubiquitin-conjugating enzyme, isoform D (Drosophila melanogaster)	26.5	67.5	76%	1.0	69%	NP_001262462.1
BIR repeat containing ubiquitin-conjugating enzyme, isoform A (Drosophila melanogaster)	26.5	67.5	76%	1.0	69%	NP_649995.2
BIR repeat containing ubiquitin-conjugating enzyme, isoform B (Drosophila melanogaster)	26.5	67.5	76%	1.0	69%	NP_001262460.1
uncharacterized protein Dmel_CG17109, isoform A (Drosophila melanogaster)	26.1	49.0	64%	1.4	53%	NP_651122.2
Cyp309a2 (Drosophila melanogaster)	25.7	25.7	60%	1.9	53%	NP_608689.2
TER94, isoform D (Drosophila melanogaster)	25.7	25.7	32%	2.0	88%	NP_001097250.1
TER94, isoform A (Drosophila melanogaster)	25.7	25.7	32%	2.0	88%	NP_477369.1
TER94, isoform E (Drosophila melanogaster)	25.7	25.7	32%	2.0	88%	NP_001286261.1
TER94, isoform C (Drosophila melanogaster)	25.7	25.7	32%	2.0	88%	NP_001097249.1
uncharacterized protein Dmel_CG6465 (Drosophila melanogaster)	24.4	24.4	28%	5.3	86%	NP_650004.1
gustatory receptor 97a, isoform A (Drosophila melanogaster)	24.4	24.4	28%	5.3	86%	NP_788747.1

gustatory receptor 97a, isoform B (Drosophila melanogaster)	24.4	24.4	28%	5.3	86%	NP_001287551.1
skpA associated protein, isoform G (Drosophila melanogaster)	24.4	24.4	32%	5.3	75%	NP_001189198.2
skpA associated protein, isoform J (Drosophila melanogaster)	24.4	24.4	32%	5.3	75%	NP_001303475.1
skpA associated protein, isoform A (Drosophila melanogaster)	24.4	24.4	32%	5.3	75%	NP_649846.2
skpA associated protein, isoform C (Drosophila melanogaster)	24.4	24.4	32%	5.3	75%	NP_001163558.1
mitochondrial RNA polymerase, isoform A (Drosophila melanogaster)	24.4	35.2	76%	5.4	47%	NP_608565.1
uncharacterized protein Dmel_CG11384 (Drosophila melanogaster)	24.0	49.8	76%	7.5	64%	NP_569887.1
uncharacterized protein Dmel_CG15394, isoform C (Drosophila melanogaster)	23.5	23.5	36%	10	67%	NP_001097062.2
uncharacterized protein Dmel_CG9485, isoform A (Drosophila melanogaster)	23.5	54.1	68%	11	62%	NP_726061.1
uncharacterized protein Dmel_CG9485, isoform C (Drosophila melanogaster)	23.5	54.1	68%	11	62%	NP_611577.2
uncharacterized protein Dmel_CG9485, isoform D (Drosophila melanogaster)	23.5	54.1	68%	11	62%	NP_001163234.1
uncharacterized protein Dmel_CG9485, isoform B (Drosophila melanogaster)	23.5	54.1	68%	11	62%	NP_726062.2
debris buster, isoform G (Drosophila melanogaster)	23.1	23.1	36%	14	70%	NP_001261299.1
transformer 2, isoform E (Drosophila melanogaster)	23.1	23.1	36%	14	67%	NP_476766.1
transformer 2, isoform C (Drosophila melanogaster)	23.1	23.1	36%	14	67%	NP_476765.1
transformer 2, isoform A (Drosophila melanogaster)	23.1	23.1	36%	14	67%	NP_476764.1
taspase 1, isoform A (Drosophila melanogaster)	23.1	23.1	32%	15	75%	NP_648832.1
phosphatase and tensin homolog, isoform C (Drosophila melanogaster)	23.1	36.1	52%	15	53%	NP_477424.1
phosphatase and tensin homolog, isoform A (Drosophila melanogaster)	23.1	36.1	52%	15	53%	NP_599147.1
phosphatase and tensin homolog, isoform B (Drosophila melanogaster)	23.1	36.1	52%	15	53%	NP_477423.1
Smg5, isoform A (Drosophila melanogaster)	23.1	23.1	60%	15	47%	NP_609685.1
wings up A, isoform K (Drosophila melanogaster)	22.7	22.7	52%	20	50%	NP_001259668.1
wings up A, isoform J (Drosophila melanogaster)	22.7	22.7	52%	20	50%	NP_001245734.1
wings up A, isoform A (Drosophila melanogaster)	22.7	22.7	52%	20	50%	NP_728141.1
uncharacterized protein Dmel_CG18327, isoform A (Drosophila melanogaster)	22.7	22.7	32%	20	75%	NP_610934.1
uncharacterized protein Dmel_CG14507 (Drosophila melanogaster)	22.7	22.7	32%	20	78%	NP_001097964.1
uncharacterized protein Dmel_CG6178 (Drosophila melanogaster)	22.7	22.7	24%	20	100%	NP_651221.1
dim gamma-tubulin 3 (Drosophila melanogaster)	22.7	22.7	36%	20	67%	NP_611533.2

uncharacterized protein Dmel_CG9934, isoform A (Drosophila melanogaster)	22.7	35.2	56%	21	67%	NP_609597.1
Muscle-specific protein 300 kDa, isoform B (Drosophila melanogaster)	22.7	113	80%	21	41%	NP_001188692.1
Muscle-specific protein 300 kDa, isoform K (Drosophila melanogaster)	22.7	140	88%	21	41%	NP_001260089.1
Muscle-specific protein 300 kDa, isoform H (Drosophila melanogaster)	22.7	140	88%	21	41%	NP_001188696.1
Muscle-specific protein 300 kDa, isoform I (Drosophila melanogaster)	22.7	140	88%	21	41%	NP_001188697.1
Muscle-specific protein 300 kDa, isoform L (Drosophila melanogaster)	22.7	156	88%	21	41%	NP_001260090.1
Muscle-specific protein 300 kDa, isoform D (Drosophila melanogaster)	22.7	156	88%	21	41%	NP_001188694.1
uncharacterized protein Dmel_CG12123 (Drosophila melanogaster)	22.3	22.3	20%	28	100%	NP_572478.1
gustatory receptor 68a (Drosophila melanogaster)	22.3	22.3	20%	28	100%	NP_524027.2
Cyp9h1 (Drosophila melanogaster)	22.3	22.3	32%	29	75%	NP_610820.1
no-on-and-no-off transient C, isoform A (Drosophila melanogaster)	22.3	33.9	40%	29	100%	NP_727132.1
accessory gland protein 26Aa (Drosophila melanogaster)	21.8	21.8	52%	39	43%	NP_476644.1
sucb, isoform A (Drosophila melanogaster)	21.8	21.8	32%	40	63%	NP_651963.1
Cyp12c1, isoform A (Drosophila melanogaster)	21.8	21.8	24%	40	83%	NP_649052.1
spookier (Drosophila melanogaster)	21.8	21.8	36%	40	78%	NP_001104460.2
uncharacterized protein Dmel_CG17803, isoform A (Drosophila melanogaster)	21.8	36.1	40%	40	56%	NP_650657.2
torn and diminished rhabdomeres, isoform C (Drosophila melanogaster)	21.8	37.3	48%	40	47%	NP_610082.2
torn and diminished rhabdomeres, isoform D (Drosophila melanogaster)	21.8	37.3	48%	40	47%	NP_724308.2
Netrin-B, isoform G (Drosophila melanogaster)	21.8	21.8	28%	40	71%	NP_001259546.1
Netrin-B, isoform A (Drosophila melanogaster)	21.8	21.8	28%	40	71%	NP_511155.1
clathrin heavy chain, isoform A (Drosophila melanogaster)	21.8	93.3	72%	40	47%	NP_477042.1
paralytic, isoform BH (Drosophila melanogaster)	21.8	34.8	96%	40	43%	NP_001285333.1
paralytic, isoform BD (Drosophila melanogaster)	21.8	34.8	96%	40	43%	NP_001188651.1
paralytic, isoform AT (Drosophila melanogaster)	21.8	34.8	96%	40	43%	NP_001188641.1
paralytic, isoform AW (Drosophila melanogaster)	21.8	34.8	96%	40	43%	NP_001188644.1
paralytic, isoform BB (Drosophila melanogaster)	21.8	34.8	96%	40	43%	NP_001188649.1
paralytic, isoform AS (Drosophila melanogaster)	21.8	34.8	96%	40	43%	NP_001188640.1
paralytic, isoform Q (Drosophila melanogaster)	21.8	34.8	96%	40	43%	NP_001188612.1

paralytic, isoform K (Drosophila melanogaster)	21.8	34.8	96%	40	43%	NP_001188606.1
paralytic, isoform R (Drosophila melanogaster)	21.8	34.8	96%	40	43%	NP_001188613.1
paralytic, isoform I (Drosophila melanogaster)	21.8	34.8	96%	40	43%	NP_001188604.1
paralytic, isoform AM (Drosophila melanogaster)	21.8	34.8	96%	40	43%	NP_001188634.1
paralytic, isoform AA (Drosophila melanogaster)	21.8	34.8	96%	40	43%	NP_001188622.1
paralytic, isoform AI (Drosophila melanogaster)	21.8	34.8	96%	40	43%	NP_001188630.1
paralytic, isoform AD (Drosophila melanogaster)	21.8	34.8	96%	40	43%	NP_001188625.1
paralytic, isoform AB (Drosophila melanogaster)	21.8	34.8	96%	40	43%	NP_001188623.1
paralytic, isoform L (Drosophila melanogaster)	21.8	34.8	96%	40	43%	NP_001188607.1
paralytic, isoform G (Drosophila melanogaster)	21.8	34.8	96%	40	43%	NP_001188602.1
paralytic, isoform AQ (Drosophila melanogaster)	21.8	34.8	96%	40	43%	NP_001188638.1
paralytic, isoform AG (Drosophila melanogaster)	21.8	34.8	96%	40	43%	NP_001188628.1
paralytic, isoform Z (Drosophila melanogaster)	21.8	34.8	96%	40	43%	NP_001188621.1
paralytic, isoform O (Drosophila melanogaster)	21.8	34.8	96%	40	43%	NP_001188610.1
paralytic, isoform AP (Drosophila melanogaster)	21.8	34.8	96%	40	43%	NP_001188637.1
paralytic, isoform V (Drosophila melanogaster)	21.8	34.8	96%	40	43%	NP_001188617.1
paralytic, isoform H (Drosophila melanogaster)	21.8	34.8	96%	40	43%	NP_001188603.1
paralytic, isoform AX (Drosophila melanogaster)	21.8	34.8	96%	40	43%	NP_001188645.1
paralytic, isoform S (Drosophila melanogaster)	21.8	34.8	96%	40	43%	NP_001188614.1
paralytic, isoform N (Drosophila melanogaster)	21.8	34.8	96%	40	43%	NP_001188609.1
paralytic, isoform M (Drosophila melanogaster)	21.8	34.8	96%	40	43%	NP_001188608.1
paralytic, isoform J (Drosophila melanogaster)	21.8	34.8	96%	40	43%	NP_001188605.1
paralytic, isoform W (Drosophila melanogaster)	21.8	34.8	96%	40	43%	NP_001188618.1
paralytic, isoform AH (Drosophila melanogaster)	21.8	34.8	96%	40	43%	NP_001188629.1
paralytic, isoform AE (Drosophila melanogaster)	21.8	34.8	96%	40	43%	NP_001188626.1
paralytic, isoform F (Drosophila melanogaster)	21.8	34.8	96%	40	43%	NP_001188601.1
paralytic, isoform E (Drosophila melanogaster)	21.8	34.8	96%	40	43%	NP_001188600.1
paralytic, isoform AF (Drosophila melanogaster)	21.8	34.8	96%	40	43%	NP_001188627.1

paralytic, isoform T (Drosophila melanogaster)	21.8	34.8	96%	40	43%	NP_001188615.1
paralytic, isoform AZ (Drosophila melanogaster)	21.8	34.8	96%	40	43%	NP_001188647.1
paralytic, isoform AL (Drosophila melanogaster)	21.8	34.8	96%	40	43%	NP_001188633.1
paralytic, isoform AO (Drosophila melanogaster)	21.8	34.8	96%	40	43%	NP_001188636.1
paralytic, isoform D (Drosophila melanogaster)	21.8	34.8	96%	40	43%	NP_001138205.1
uncharacterized protein Dmel_CG9674, isoform A (Drosophila melanogaster)	21.8	36.9	44%	40	62%	NP_648922.1
uncharacterized protein Dmel_CG9674, isoform F (Drosophila melanogaster)	21.8	36.9	44%	40	62%	NP_001246804.1
paralytic, isoform U (Drosophila melanogaster)	21.8	34.8	96%	40	43%	NP_001188616.1
paralytic, isoform P (Drosophila melanogaster)	21.8	34.8	96%	40	43%	NP_001188611.1
paralytic, isoform X (Drosophila melanogaster)	21.8	34.8	96%	40	43%	NP_001188619.1
paralytic, isoform A (Drosophila melanogaster)	21.8	34.8	96%	40	43%	NP_523371.2
paralytic, isoform C (Drosophila melanogaster)	21.8	34.8	96%	40	43%	NP_001036281.1
paralytic, isoform B (Drosophila melanogaster)	21.8	34.8	96%	40	43%	NP_001036280.1
paralytic, isoform BA (Drosophila melanogaster)	21.8	34.8	96%	40	43%	NP_001188648.1
paralytic, isoform AY (Drosophila melanogaster)	21.8	34.8	96%	40	43%	NP_001188646.1
paralytic, isoform AJ (Drosophila melanogaster)	21.8	34.8	96%	40	43%	NP_001188631.1
paralytic, isoform Y (Drosophila melanogaster)	21.8	34.8	96%	40	43%	NP_001188620.1
paralytic, isoform AN (Drosophila melanogaster)	21.8	34.8	96%	40	43%	NP_001188635.1
paralytic, isoform BF (Drosophila melanogaster)	21.8	34.8	96%	40	43%	NP_001259619.1
ubiquitin conjugating enzyme 4, isoform A (Drosophila melanogaster)	21.4	21.4	32%	54	63%	NP_524010.2
uncharacterized protein Dmel_CG31207 (Drosophila melanogaster)	21.4	53.7	88%	55	55%	NP_732581.1
cuticular protein 49Ac, isoform E (Drosophila melanogaster)	21.4	21.4	28%	55	86%	NP_001286344.1
cuticular protein 49Ac, isoform A (Drosophila melanogaster)	21.4	21.4	28%	55	86%	NP_610772.3
uncharacterized protein Dmel_CG8031 (Drosophila melanogaster)	21.4	21.4	28%	55	86%	NP_650252.1
cuticular protein 49Ac, isoform F (Drosophila melanogaster)	21.4	21.4	28%	55	86%	NP_001286345.1
cuticular protein 49Ac, isoform C (Drosophila melanogaster)	21.4	21.4	28%	55	86%	NP_725150.2
uncharacterized protein Dmel_CG5618, isoform E (Drosophila melanogaster)	21.4	21.4	76%	55	35%	NP_996124.1
mitochondrial transcription termination factor 3 (Drosophila melanogaster)	21.4	21.4	28%	55	71%	NP_649240.1

rhodopsin 2 (Drosophila melanogaster)	21.4	21.4	40%	55	60%	NP_524398.1
Cyp28a5 (Drosophila melanogaster)	21.4	21.4	36%	56	56%	NP_609694.1
uncharacterized protein Dmel_CG9784, isoform A (Drosophila melanogaster)	21.4	21.4	80%	56	39%	NP_573135.1
uncharacterized protein Dmel_CG5618, isoform A (Drosophila melanogaster)	21.4	21.4	76%	56	35%	NP_649211.1
uncharacterized protein Dmel_CG9784, isoform C (Drosophila melanogaster)	21.4	21.4	80%	56	39%	NP_001259623.1
amun, isoform C (Drosophila melanogaster)	21.4	21.4	52%	56	53%	NP_572741.1
withered, isoform A (Drosophila melanogaster)	21.4	21.4	20%	56	100%	NP_523685.2
withered, isoform C (Drosophila melanogaster)	21.4	21.4	20%	56	100%	NP_001163111.1
female sterile (1) M3 (Drosophila melanogaster)	21.4	39.0	44%	56	100%	NP_572284.4
uncharacterized protein Dmel_CG32163, isoform B (Drosophila melanogaster)	21.0	21.0	52%	68	62%	NP_001287083.1
uncharacterized protein Dmel_CG42615 (Drosophila melanogaster)	21.0	21.0	48%	69	56%	NP_001163091.1
uncharacterized protein Dmel_CG32163, isoform A (Drosophila melanogaster)	21.0	21.0	52%	74	62%	NP_730181.1
uncharacterized protein Dmel_CG44014 (Drosophila melanogaster)	21.0	21.0	60%	76	53%	NP_732043.1
cyclin J, isoform B (Drosophila melanogaster)	21.0	31.8	68%	76	43%	NP_995989.1
uncharacterized protein Dmel_CG11980, isoform A (Drosophila melanogaster)	21.0	21.0	32%	77	75%	NP_731303.2
uncharacterized protein Dmel_CG11980, isoform B (Drosophila melanogaster)	21.0	21.0	32%	77	75%	NP_731302.1
uncharacterized protein Dmel_CG11980, isoform C (Drosophila melanogaster)	21.0	21.0	32%	77	75%	NP_649857.1
yellow-e2 (Drosophila melanogaster)	21.0	21.0	72%	77	50%	NP_650289.2
uncharacterized protein Dmel_CG7639 (Drosophila melanogaster)	21.0	21.0	44%	77	67%	NP_995838.1
uncharacterized protein Dmel_CG12910, isoform B (Drosophila melanogaster)	21.0	21.0	20%	78	100%	NP_001260857.1
uncharacterized protein Dmel_CG12910, isoform A (Drosophila melanogaster)	21.0	21.0	20%	78	100%	NP_610569.1
rasp (Drosophila melanogaster)	21.0	21.0	24%	78	75%	NP_523898.1
uncharacterized protein Dmel_CG2145, isoform A (Drosophila melanogaster)	21.0	21.0	36%	78	47%	NP_572668.1
reduced ocelli, isoform A (Drosophila melanogaster)	21.0	37.3	56%	78	56%	NP_609861.1
reduced ocelli, isoform B (Drosophila melanogaster)	21.0	37.3	56%	78	56%	NP_001286024.1
WD repeat domain 33, isoform A (Drosophila melanogaster)	21.0	21.0	24%	78	83%	NP_730982.1
uncharacterized protein Dmel_CG31533 (Drosophila melanogaster)	21.0	33.9	52%	78	75%	NP_731887.1
RNA polymerase III 128kD subunit (Drosophila melanogaster)	21.0	31.8	60%	78	43%	NP_523706.1

CAP-D2 condensin subunit (Drosophila melanogaster)	21.0	21.0	20%	78	100%	NP_651709.1
uncharacterized protein Dmel_CG8771, isoform B (Drosophila melanogaster)	21.0	70.2	96%	79	55%	NP_610810.2
uncharacterized protein Dmel_CG8771, isoform C (Drosophila melanogaster)	21.0	70.2	96%	79	55%	NP_001246283.1
skuld, isoform F (Drosophila melanogaster)	21.0	61.9	76%	79	71%	NP_001262134.1
skuld, isoform D (Drosophila melanogaster)	21.0	61.9	76%	79	71%	NP_524653.1
skuld, isoform E (Drosophila melanogaster)	21.0	61.9	76%	79	71%	NP_730591.2
uncharacterized protein Dmel_CG33690 (Drosophila melanogaster)	20.6	20.6	20%	105	100%	NP_001027133.1
synaptotagmin 7, isoform F (Drosophila melanogaster)	20.6	31.4	56%	107	58%	NP_995593.2
uncharacterized protein Dmel_CG14830, isoform A (Drosophila melanogaster)	20.6	20.6	32%	107	67%	NP_648095.2
synaptotagmin 7, isoform B (Drosophila melanogaster)	20.6	31.4	56%	107	58%	NP_726558.2
Ucp4A, isoform A (Drosophila melanogaster)	20.6	20.6	24%	107	83%	NP_573246.1
uncharacterized protein Dmel_CG31785 (Drosophila melanogaster)	20.6	36.9	52%	108	35%	NP_724093.2
synaptotagmin 7, isoform A (Drosophila melanogaster)	20.6	31.4	56%	108	58%	NP_726557.3
uncharacterized protein Dmel_CG3734, isoform A (Drosophila melanogaster)	20.6	35.6	36%	108	100%	NP_650802.1
cytochrome P450-4e2, isoform A (Drosophila melanogaster)	20.6	20.6	32%	108	70%	NP_477117.2
kinetochore and EB1 associated basic protein, isoform A (Drosophila melanogaster)	20.6	20.6	52%	108	62%	NP_722756.1
kinetochore and EB1 associated basic protein, isoform B (Drosophila melanogaster)	20.6	20.6	52%	108	62%	NP_001259900.1
uncharacterized protein Dmel_CG2162, isoform E (Drosophila melanogaster)	20.6	20.6	36%	109	50%	NP_001163332.2
uncharacterized protein Dmel_CG10483 (Drosophila melanogaster)	20.6	36.9	68%	109	53%	NP_648000.1
Threonyl-tRNA synthetase, isoform C (Drosophila melanogaster)	20.6	55.4	56%	109	71%	NP_723726.1
Acyl-CoA synthetase long-chain, isoform D (Drosophila melanogaster)	20.6	34.4	64%	109	47%	NP_001014512.1
brivido-3, isoform B (Drosophila melanogaster)	20.6	20.6	24%	109	83%	NP_570013.3
Threonyl-tRNA synthetase, isoform A (Drosophila melanogaster)	20.6	55.4	56%	109	71%	NP_723725.1
brivido-3, isoform C (Drosophila melanogaster)	20.6	20.6	24%	109	83%	NP_001284831.1
sirtuin 7 (Drosophila melanogaster)	20.6	34.8	56%	109	70%	NP_651664.2
Ecdysone-induced protein 74EF, isoform A (Drosophila melanogaster)	20.6	20.6	20%	109	100%	NP_730287.1
Ecdysone-induced protein 74EF, isoform E (Drosophila melanogaster)	20.6	20.6	20%	109	100%	NP_001246816.1
Ecdysone-induced protein 74EF, isoform C (Drosophila melanogaster)	20.6	20.6	20%	109	100%	NP_730286.3

Ecdysone-induced protein 74EF, isoform B (Drosophila melanogaster)	20.6	33.1	40%	109	100%	NP_730288.1
spineless, isoform A (Drosophila melanogaster)	20.6	20.6	52%	109	54%	NP_476748.1
uncharacterized protein Dmel_CG42335 (Drosophila melanogaster)	20.6	20.6	76%	109	48%	NP_732655.3
uncharacterized protein Dmel_CG31445, isoform A (Drosophila melanogaster)	20.6	31.4	44%	109	67%	NP_651689.1
uncharacterized protein Dmel_CG2111 (Drosophila melanogaster)	20.6	48.2	64%	109	67%	NP_572644.1
spineless, isoform C (Drosophila melanogaster)	20.6	20.6	52%	109	54%	NP_001247140.1
uncharacterized protein Dmel_CG33158 (Drosophila melanogaster)	20.6	33.1	24%	109	67%	NP_788515.1
histone gene-specific epigenetic repressor in late S phase, isoform H (Drosophila melanogaster)	20.6	20.6	40%	109	47%	NP_001245774.1
pyruvate carboxylase, isoform E (Drosophila melanogaster)	20.6	20.6	32%	109	75%	NP_610527.1
pyruvate carboxylase, isoform I (Drosophila melanogaster)	20.6	20.6	32%	109	75%	NP_724841.1
histone gene-specific epigenetic repressor in late S phase, isoform C (Drosophila melanogaster)	20.6	20.6	40%	109	47%	NP_728309.1
histone gene-specific epigenetic repressor in late S phase, isoform D (Drosophila melanogaster)	20.6	35.2	56%	109	47%	NP_001033855.1
defective transmitter release (Drosophila melanogaster)	20.6	43.5	32%	109	100%	NP_724335.2
molting defective, isoform F (Drosophila melanogaster)	20.6	35.2	24%	110	83%	NP_001247288.1
molting defective, isoform A (Drosophila melanogaster)	20.6	35.2	24%	110	83%	NP_001036754.1
molting defective, isoform E (Drosophila melanogaster)	20.6	35.2	24%	110	83%	NP_651273.2
molting defective, isoform D (Drosophila melanogaster)	20.6	35.2	24%	110	83%	NP_001247289.1
histone gene-specific epigenetic repressor in late S phase, isoform A (Drosophila melanogaster)	20.6	31.4	40%	110	47%	NP_608360.2
Unc-89, isoform G (Drosophila melanogaster)	20.6	49.0	56%	110	83%	NP_001286805.1
uninflatable, isoform B (Drosophila melanogaster)	20.6	76.2	72%	110	71%	NP_001137804.1
uninflatable, isoform C (Drosophila melanogaster)	20.6	76.2	72%	110	71%	NP_001162899.1
Unc-89, isoform H (Drosophila melanogaster)	20.6	63.2	64%	110	83%	NP_001286806.1
Unc-89, isoform F (Drosophila melanogaster)	20.6	49.0	56%	110	83%	NP_001286804.1
Unc-89, isoform E (Drosophila melanogaster)	20.6	63.2	64%	110	83%	NP_001286803.1
Unc-89, isoform D (Drosophila melanogaster)	20.6	63.2	64%	110	83%	NP_001014545.3
Unc-89, isoform C (Drosophila melanogaster)	20.6	63.2	64%	110	83%	NP_001097440.1
male fertility factor kl3 (Drosophila melanogaster)	20.6	126	76%	110	53%	NP_001104482.3
Ran, isoform A (Drosophila melanogaster)	20.2	20.2	56%	147	50%	NP_651969.1

glutathione S transferase E1 (Drosophila melanogaster)	20.2	20.2	32%	148	67%	NP_611323.1
uncharacterized protein Dmel_CG17744 (Drosophila melanogaster)	20.2	31.0	56%	148	64%	NP_648070.1
uncharacterized protein Dmel_CG15880 (Drosophila melanogaster)	20.2	20.2	24%	149	55%	NP_608547.2
mei-217 (Drosophila melanogaster)	20.2	20.2	20%	149	100%	NP_001027069.3
GlcAT-P, isoform C (Drosophila melanogaster)	20.2	35.2	56%	149	47%	NP_001014581.1
uncharacterized protein Dmel_CG6048 (Drosophila melanogaster)	20.2	20.2	36%	150	62%	NP_572282.1
uncharacterized protein Dmel_CG5955 (Drosophila melanogaster)	20.2	20.2	28%	150	58%	NP_649230.1
gustatory receptor 39a, isoform B (Drosophila melanogaster)	20.2	31.8	24%	150	100%	NP_724329.1
uncharacterized protein Dmel_CG17110, isoform A (Drosophila melanogaster)	20.2	20.2	28%	150	71%	NP_651121.1
uncharacterized protein Dmel_CG5316, isoform E (Drosophila melanogaster)	20.2	20.2	24%	150	83%	NP_001247195.2
uncharacterized protein Dmel_CG2129 (Drosophila melanogaster)	20.2	20.2	32%	151	67%	NP_572449.1
uncharacterized protein Dmel_CG18472 (Drosophila melanogaster)	20.2	20.2	24%	151	75%	NP_651514.1
GlcAT-P, isoform A (Drosophila melanogaster)	20.2	51.1	56%	151	47%	NP_648448.1
uncharacterized protein Dmel_CG18095, isoform A (Drosophila melanogaster)	20.2	31.8	44%	151	64%	NP_609697.2
sensory neuron membrane protein 1, isoform A (Drosophila melanogaster)	20.2	31.0	48%	151	100%	NP_650953.1
uncharacterized protein Dmel_CG18095, isoform B (Drosophila melanogaster)	20.2	46.0	44%	151	64%	NP_001188805.1
uncharacterized protein Dmel_CG5316, isoform B (Drosophila melanogaster)	20.2	20.2	24%	151	83%	NP_650805.1
TBC1 domain family member 16 (Drosophila melanogaster)	20.2	20.2	28%	151	71%	NP_609403.2
uncharacterized protein Dmel_CG16854, isoform B (Drosophila melanogaster)	20.2	20.2	36%	151	70%	NP_001245974.1
Na(+)/H(+) hydrogen antiporter 1, isoform B (Drosophila melanogaster)	20.2	31.8	52%	151	71%	NP_609080.1
Na(+)/H(+) hydrogen antiporter 1, isoform A (Drosophila melanogaster)	20.2	31.8	52%	151	71%	NP_723224.2
mediator complex subunit 15 (Drosophila melanogaster)	20.2	20.2	40%	152	58%	NP_608528.1
uncharacterized protein Dmel_CG6424, isoform A (Drosophila melanogaster)	20.2	31.4	28%	152	58%	NP_725709.1
gemini, isoform A (Drosophila melanogaster)	20.2	49.0	76%	152	33%	NP_724895.1
gemini, isoform C (Drosophila melanogaster)	20.2	49.0	76%	152	33%	NP_610556.1
stall, isoform C (Drosophila melanogaster)	20.2	20.2	20%	152	100%	NP_001097423.1
uncharacterized protein Dmel_CG4329 (Drosophila melanogaster)	20.2	32.7	56%	152	70%	NP_611687.1
Kinesin-like protein at 98A, isoform B (Drosophila melanogaster)	20.2	36.1	44%	152	83%	NP_001247339.1

Kinesin-like protein at 98A, isoform A (Drosophila melanogaster)	20.2	50.3	60%	152	83%	NP_524532.2
integrator 6, isoform A (Drosophila melanogaster)	20.2	34.8	36%	152	100%	NP_572253.2
cacophony, isoform M (Drosophila melanogaster)	20.2	32.2	60%	153	56%	NP_001245637.1
cacophony, isoform B (Drosophila melanogaster)	20.2	32.2	60%	153	56%	NP_996420.1
cacophony, isoform D (Drosophila melanogaster)	20.2	32.2	60%	153	56%	NP_996418.1
cacophony, isoform C (Drosophila melanogaster)	20.2	32.2	60%	153	56%	NP_996419.1
cacophony, isoform A (Drosophila melanogaster)	20.2	32.2	60%	153	56%	NP_511133.2
cacophony, isoform H (Drosophila melanogaster)	20.2	32.2	60%	153	56%	NP_001014734.1
cacophony, isoform G (Drosophila melanogaster)	20.2	32.2	60%	153	56%	NP_001014735.1
cacophony, isoform I (Drosophila melanogaster)	20.2	32.2	60%	153	56%	NP_001014733.1

Table S2. TOP 250 Proteins that could be detected by the antibody (against aa1-80) used in Ni et al., [20].

Description	Max Score	Total Score	Query Cover	E Value	Ident	Accession
rhodopsin 7 (Drosophila melanogaster)	247	247	100%	3e-74	100%	NP_524035.2
activating transcription factor 3, isoform A (Drosophila melanogaster)	33.3	107	53%	0.071	67%	NP_620473.1
activating transcription factor 3, isoform B (Drosophila melanogaster)	33.3	107	53%	0.072	67%	NP_001259125.1
SAM-motif ubiquitously expressed punctatedly localized protein, isoform D (Drosophila melanogaster)	32.0	308	30%	0.18	47%	NP_001285828.1
SAM-motif ubiquitously expressed punctatedly localized protein, isoform A (Drosophila melanogaster)	32.0	368	46%	0.18	47%	NP_525101.2
uncharacterized protein Dmel_CG13946 (Drosophila melanogaster)	31.6	31.6	28%	0.19	57%	NP_652360.1
uncharacterized protein Dmel_CG14650 (Drosophila melanogaster)	31.2	73.8	51%	0.33	58%	NP_649473.1
uncharacterized protein Dmel_CG5756, isoform C (Drosophila melanogaster)	31.2	192	63%	0.33	75%	NP_611292.2
protein phosphatase 2C, isoform A (Drosophila melanogaster)	30.8	175	38%	0.45	54%	NP_525074.2
connectin, isoform A (Drosophila melanogaster)	29.9	75.1	56%	0.81	43%	NP_523930.3
stranded at second, isoform A (Drosophila melanogaster)	29.9	219	68%	0.83	54%	NP_476611.1
stranded at second, isoform B (Drosophila melanogaster)	29.9	238	68%	0.83	54%	NP_731141.1
uncharacterized protein Dmel_CG18304, isoform C (Drosophila melanogaster)	29.9	162	70%	0.83	77%	NP_001260169.1
uncharacterized protein Dmel_CG18304, isoform B (Drosophila melanogaster)	29.9	162	70%	0.83	77%	NP_001245921.1
uncharacterized protein Dmel_CG18304, isoform A (Drosophila melanogaster)	29.9	162	70%	0.83	77%	NP_609083.2

uncharacterized protein Dmel_CG18304, isoform D (Drosophila melanogaster)	29.9	162	70%	0.83	77%	NP_001260170.1
multiple ankyrin repeats single KH domain, isoform F (Drosophila melanogaster)	29.9	355	80%	0.84	62%	NP_001287500.1
multiple ankyrin repeats single KH domain, isoform D (Drosophila melanogaster)	29.9	355	80%	0.84	62%	NP_001262903.1
multiple ankyrin repeats single KH domain, isoform E (Drosophila melanogaster)	29.9	516	83%	0.84	62%	NP_001262904.1
multiple ankyrin repeats single KH domain, isoform A (Drosophila melanogaster)	29.9	516	83%	0.84	62%	NP_788733.1
multiple ankyrin repeats single KH domain, isoform C (Drosophila melanogaster)	29.9	516	83%	0.84	62%	NP_001247280.1
uncharacterized protein Dmel_CG34426 (Drosophila melanogaster)	29.5	93.1	77%	1.1	45%	NP_001097552.1
pointed, isoform C (Drosophila melanogaster)	29.5	151	62%	1.1	67%	NP_732858.1
pointed, isoform E (Drosophila melanogaster)	29.5	151	62%	1.1	67%	NP_001262872.1
hormone receptor 4, isoform C (Drosophila melanogaster)	29.5	311	53%	1.1	52%	NP_001033823.1
hormone receptor 4, isoform D (Drosophila melanogaster)	29.5	311	53%	1.1	52%	NP_001259159.1
hormone receptor 4, isoform F (Drosophila melanogaster)	29.5	311	53%	1.1	52%	NP_001259157.1
hormone receptor 4, isoform E (Drosophila melanogaster)	29.5	335	53%	1.1	52%	NP_001259158.1
hormone receptor 4, isoform J (Drosophila melanogaster)	29.5	335	53%	1.1	52%	NP_001259162.1
hormone receptor 4, isoform H (Drosophila melanogaster)	29.5	381	65%	1.1	52%	NP_001259160.1
hormone receptor 4, isoform G (Drosophila melanogaster)	29.5	335	53%	1.1	52%	NP_001259156.1
hormone receptor 4, isoform I (Drosophila melanogaster)	29.5	448	66%	1.1	52%	NP_001259161.1
Muscle-specific protein 300 kDa, isoform B (Drosophila melanogaster)	29.5	497	87%	1.1	50%	NP_001188692.1
Muscle-specific protein 300 kDa, isoform L (Drosophila melanogaster)	29.5	735	91%	1.1	50%	NP_001260090.1
Muscle-specific protein 300 kDa, isoform D (Drosophila melanogaster)	29.5	748	91%	1.1	50%	NP_001188694.1
uncharacterized protein Dmel_CG13075 (Drosophila melanogaster)	29.1	174	31%	1.4	56%	NP_648831.1
uncharacterized protein Dmel_CG44838, isoform G (Drosophila melanogaster)	29.1	171	51%	1.5	52%	NP_648321.2
uncharacterized protein Dmel_CG44838, isoform I (Drosophila melanogaster)	29.1	205	65%	1.5	52%	NP_001287006.1
uncharacterized protein Dmel_CG43867, isoform I (Drosophila melanogaster)	29.1	221	61%	1.5	45%	NP_001259105.1
uncharacterized protein Dmel_CG43867, isoform L (Drosophila melanogaster)	29.1	218	61%	1.5	45%	NP_477389.4
uncharacterized protein Dmel_CG43867, isoform F (Drosophila melanogaster)	29.1	235	61%	1.5	45%	NP_001096861.1
uncharacterized protein Dmel_CG43867, isoform H (Drosophila melanogaster)	29.1	291	82%	1.5	45%	NP_001259106.1
uncharacterized protein Dmel_CG43867, isoform B (Drosophila melanogaster)	29.1	237	67%	1.5	45%	NP_001259108.1

uncharacterized protein Dmel_CG43867, isoform A (Drosophila melanogaster)	29.1	254	67%	1.5	45%	NP_001259109.1
uncharacterized protein Dmel_CG43867, isoform D (Drosophila melanogaster)	29.1	321	82%	1.5	45%	NP_001096860.2
uncharacterized protein Dmel_CG43867, isoform C (Drosophila melanogaster)	29.1	251	67%	1.5	45%	NP_001259104.1
uncharacterized protein Dmel_CG43867, isoform K (Drosophila melanogaster)	29.1	268	67%	1.5	45%	NP_001284762.1
mucin related 29B (Drosophila melanogaster)	28.6	435	60%	2.0	37%	NP_723377.1
acinus, isoform A (Drosophila melanogaster)	28.6	56.6	31%	2.0	63%	NP_609935.1
uncharacterized protein Dmel_CG12506 (Drosophila melanogaster)	28.2	28.2	28%	2.3	52%	NP_608541.1
uncharacterized protein Dmel_CG34273 (Drosophila melanogaster)	28.2	113	30%	2.4	57%	NP_001097790.1
la costa (Drosophila melanogaster)	28.2	85.7	23%	2.4	63%	NP_523436.1
uncharacterized protein Dmel_CG13560, isoform A (Drosophila melanogaster)	28.2	196	27%	2.5	60%	NP_611823.2
uncharacterized protein Dmel_CG10911 (Drosophila melanogaster)	28.2	236	77%	2.7	48%	NP_611286.1
protein kinase, cAMP-dependent, catalytic subunit 3, isoform A (Drosophila melanogaster)	28.2	57.5	47%	2.7	39%	NP_524097.2
protein kinase, cAMP-dependent, catalytic subunit 3, isoform B (Drosophila melanogaster)	28.2	70.8	47%	2.7	39%	NP_730083.2
RNA-binding protein 9, isoform J (Drosophila melanogaster)	28.2	155	38%	2.7	52%	NP_001259974.1
uncharacterized protein Dmel_CG15725 (Drosophila melanogaster)	28.2	170	48%	2.7	43%	NP_572823.1
stubble, isoform A (Drosophila melanogaster)	28.2	105	35%	2.7	64%	NP_476709.1
wacky, isoform C (Drosophila melanogaster)	28.2	158	50%	2.8	50%	NP_001097008.2
wacky, isoform A (Drosophila melanogaster)	28.2	158	50%	2.8	50%	NP_573187.2
wacky, isoform E (Drosophila melanogaster)	28.2	158	50%	2.8	50%	NP_001259635.1
wacky, isoform G (Drosophila melanogaster)	28.2	158	50%	2.8	50%	NP_001259637.1
wacky, isoform F (Drosophila melanogaster)	28.2	173	50%	2.8	50%	NP_001259636.1
meiotic P26, isoform A (Drosophila melanogaster)	28.2	91.0	76%	2.8	30%	NP_652022.1
meiotic P26, isoform C (Drosophila melanogaster)	28.2	91.0	76%	2.8	30%	NP_001259377.1
uncharacterized protein Dmel_CG15570 (Drosophila melanogaster)	28.2	355	67%	2.8	59%	NP_572154.1
fat facets, isoform C (Drosophila melanogaster)	28.2	181	58%	2.8	48%	NP_733455.1
fat facets, isoform E (Drosophila melanogaster)	28.2	181	58%	2.8	48%	NP_001247388.1
fat facets, isoform D (Drosophila melanogaster)	28.2	219	58%	2.8	48%	NP_001247387.1
fat facets, isoform A (Drosophila melanogaster)	28.2	219	58%	2.8	48%	NP_524612.2

Ptx1, isoform A (Drosophila melanogaster)	27.8	116	53%	3.7	54%	NP_733410.2
Ptx1, isoform C (Drosophila melanogaster)	27.8	116	53%	3.7	54%	NP_996314.1
Ptx1, isoform E (Drosophila melanogaster)	27.8	133	53%	3.7	54%	NP_001138130.2
mucin 68D (Drosophila melanogaster)	27.8	1274	78%	3.8	43%	NP_648504.2
uncharacterized protein Dmel_CG17839, isoform B (Drosophila melanogaster)	27.8	206	50%	3.8	41%	NP_996085.2
mucin related 89F, isoform C (Drosophila melanogaster)	27.8	851	77%	3.8	67%	NP_650611.2
mucin related 89F, isoform B (Drosophila melanogaster)	27.8	851	77%	3.8	67%	NP_001262662.1
papilin, isoform C (Drosophila melanogaster)	27.8	511	90%	3.8	48%	NP_001163760.1
papilin, isoform F (Drosophila melanogaster)	27.8	525	90%	3.8	48%	NP_788751.2
papilin, isoform G (Drosophila melanogaster)	27.8	511	90%	3.8	48%	NP_001163761.1
papilin, isoform E (Drosophila melanogaster)	27.8	525	90%	3.8	48%	NP_788752.2
HECT and RLD domain containing protein 2 (Drosophila melanogaster)	27.8	429	76%	3.8	49%	NP_608388.2
Sox21a, isoform A (Drosophila melanogaster)	27.4	59.6	31%	4.9	56%	NP_648694.1
Sox21a, isoform B (Drosophila melanogaster)	27.4	59.6	31%	4.9	56%	NP_001261827.1
head involution defective, isoform A (Drosophila melanogaster)	27.4	140	47%	4.9	50%	NP_524136.2
Usf, isoform B (Drosophila melanogaster)	27.4	233	66%	4.9	50%	NP_572167.3
CIN85 and CD2AP related, isoform B (Drosophila melanogaster)	27.4	185	53%	5.0	67%	NP_651827.1
CIN85 and CD2AP related, isoform A (Drosophila melanogaster)	27.4	185	53%	5.0	67%	NP_733406.1
CIN85 and CD2AP related, isoform D (Drosophila melanogaster)	27.4	188	53%	5.0	67%	NP_733407.2
uncharacterized protein Dmel_CG9003, isoform B (Drosophila melanogaster)	27.4	84.2	50%	5.0	47%	NP_001097271.1
CIN85 and CD2AP related, isoform F (Drosophila melanogaster)	27.4	188	53%	5.0	67%	NP_001263125.1
CIN85 and CD2AP related, isoform G (Drosophila melanogaster)	27.4	188	53%	5.0	67%	NP_001263126.1
uncharacterized protein Dmel_CG17264, isoform A (Drosophila melanogaster)	27.4	123	87%	5.0	34%	NP_608736.2
CIN85 and CD2AP related, isoform E (Drosophila melanogaster)	27.4	229	61%	5.0	67%	NP_001263124.1
CIN85 and CD2AP related, isoform C (Drosophila melanogaster)	27.4	229	61%	5.0	67%	NP_733405.2
CIN85 and CD2AP related, isoform J (Drosophila melanogaster)	27.4	229	61%	5.1	67%	NP_001263129.1
hira (Drosophila melanogaster)	27.4	154	32%	5.1	54%	NP_572401.2
hairless, isoform B (Drosophila melanogaster)	27.4	196	73%	5.1	44%	NP_732535.1

hairless, isoform A (Drosophila melanogaster)	27.4	196	73%	5.1	44%	NP_732533.1
stardust, isoform I (Drosophila melanogaster)	27.4	73.0	35%	5.1	44%	NP_572464.4
stardust, isoform B (Drosophila melanogaster)	27.4	104	35%	5.1	44%	NP_572463.1
stardust, isoform E (Drosophila melanogaster)	27.4	104	35%	5.1	44%	NP_996376.1
stardust, isoform J (Drosophila melanogaster)	27.4	110	35%	5.1	44%	NP_001245574.1
rolling pebbles, isoform H (Drosophila melanogaster)	27.4	111	53%	5.1	43%	NP_001261738.1
rolling pebbles, isoform D (Drosophila melanogaster)	27.4	111	53%	5.1	43%	NP_648542.1
stardust, isoform O (Drosophila melanogaster)	27.4	142	35%	5.1	44%	NP_001285010.1
rolling pebbles, isoform F (Drosophila melanogaster)	27.4	111	53%	5.1	43%	NP_001261736.1
rolling pebbles, isoform C (Drosophila melanogaster)	27.4	111	53%	5.1	43%	NP_729780.1
rolling pebbles, isoform I (Drosophila melanogaster)	27.4	141	63%	5.1	43%	NP_001261739.1
rolling pebbles, isoform G (Drosophila melanogaster)	27.4	141	63%	5.1	43%	NP_001261737.1
rolling pebbles, isoform B (Drosophila melanogaster)	27.4	141	63%	5.1	43%	NP_729778.1
stardust, isoform M (Drosophila melanogaster)	27.4	304	40%	5.1	44%	NP_001259326.1
stardust, isoform L (Drosophila melanogaster)	27.4	291	40%	5.1	44%	NP_001245576.1
stardust, isoform G (Drosophila melanogaster)	27.4	323	40%	5.1	44%	NP_001033835.2
mucin 68Ca (Drosophila melanogaster)	27.4	2448	66%	5.1	54%	NP_996054.1
uncharacterized protein Dmel_CG32188 (Drosophila melanogaster)	26.9	91.8	40%	5.7	45%	NP_730308.1
uncharacterized protein Dmel_CG13054 (Drosophila melanogaster)	26.9	52.4	31%	6.6	48%	NP_648852.1
uncharacterized protein Dmel_CG6294, isoform B (Drosophila melanogaster)	26.9	42.4	35%	6.7	50%	NP_573026.1
ubiquitin specific protease 2, isoform H (Drosophila melanogaster)	26.9	105	25%	6.7	54%	NP_001285527.1
uncharacterized protein Dmel_CG13676, isoform B (Drosophila melanogaster)	26.9	56.6	23%	6.8	58%	NP_001261560.1
ubiquitin specific protease 2, isoform A (Drosophila melanogaster)	26.9	121	32%	6.8	54%	NP_608462.1
uncharacterized protein Dmel_CG13676, isoform A (Drosophila melanogaster)	26.9	72.1	28%	6.8	58%	NP_648179.3
F-box protein 11, isoform A (Drosophila melanogaster)	26.9	275	57%	6.9	50%	NP_649954.1
uncharacterized protein Dmel_CG43658, isoform D (Drosophila melanogaster)	26.9	126	50%	6.9	61%	NP_001162780.2
uncharacterized protein Dmel_CG43658, isoform C (Drosophila melanogaster)	26.9	219	78%	6.9	61%	NP_001259643.1
rhinoceros, isoform B (Drosophila melanogaster)	26.9	371	92%	6.9	36%	NP_995944.1

uncharacterized protein Dmel_CG14452 (Drosophila melanogaster)	26.5	94.6	28%	7.9	61%	NP_649407.1
uncharacterized protein Dmel_CG10918 (Drosophila melanogaster)	26.5	183	31%	8.5	48%	NP_608453.1
bunched, isoform C (Drosophila melanogaster)	26.5	42.8	25%	8.5	75%	NP_723756.1
bunched, isoform O (Drosophila melanogaster)	26.5	57.5	25%	8.5	75%	NP_001260419.1
bunched, isoform B (Drosophila melanogaster)	26.5	42.8	25%	8.6	75%	NP_001162965.1
bunched, isoform D (Drosophila melanogaster)	26.5	55.8	25%	8.7	75%	NP_001162964.1
bunched, isoform E (Drosophila melanogaster)	26.5	42.8	25%	8.7	75%	NP_001036358.1
uncharacterized protein Dmel_CG31324, isoform A (Drosophila melanogaster)	26.5	47.5	37%	8.8	47%	NP_733138.1
lemming B, isoform A (Drosophila melanogaster)	26.5	148	50%	9.0	46%	NP_001097124.1
uncharacterized protein Dmel_CG14223, isoform A (Drosophila melanogaster)	26.5	109	43%	9.1	71%	NP_608341.1
uncharacterized protein Dmel_CG46026 (Drosophila melanogaster)	26.5	880	38%	9.2	56%	NP_001303496.1
uncharacterized protein Dmel_CG10151, isoform A (Drosophila melanogaster)	26.5	117	43%	9.2	71%	NP_725414.1
uncharacterized protein Dmel_CG10151, isoform D (Drosophila melanogaster)	26.5	117	43%	9.2	71%	NP_001137670.1
simjang, isoform C (Drosophila melanogaster)	26.5	142	51%	9.3	41%	NP_648407.1
simjang, isoform A (Drosophila melanogaster)	26.5	142	51%	9.3	41%	NP_729624.1
simjang, isoform D (Drosophila melanogaster)	26.5	157	55%	9.3	41%	NP_001261684.1
simjang, isoform E (Drosophila melanogaster)	26.5	142	51%	9.3	41%	NP_001261685.1
bunched, isoform F (Drosophila melanogaster)	26.5	190	46%	9.3	75%	NP_001036359.1
bunched, isoform P (Drosophila melanogaster)	26.5	173	46%	9.3	75%	NP_001285875.1
bunched, isoform A (Drosophila melanogaster)	26.5	204	46%	9.3	75%	NP_525103.2
bunched, isoform G (Drosophila melanogaster)	26.5	204	46%	9.3	75%	NP_001036357.2
sickie, isoform K (Drosophila melanogaster)	26.5	177	68%	9.3	45%	NP_001260608.1
sickie, isoform M (Drosophila melanogaster)	26.5	205	68%	9.4	45%	NP_001260610.1
sickie, isoform D (Drosophila melanogaster)	26.5	220	70%	9.4	45%	NP_001097183.1
sickie, isoform N (Drosophila melanogaster)	26.5	205	68%	9.4	45%	NP_995730.2
sickie, isoform H (Drosophila melanogaster)	26.5	220	68%	9.4	45%	NP_001260603.1
sickie, isoform B (Drosophila melanogaster)	26.5	205	68%	9.4	45%	NP_995729.2
sickie, isoform L (Drosophila melanogaster)	26.5	205	68%	9.4	45%	NP_001260609.1

megator, isoform A (Drosophila melanogaster)	26.5	258	65%	9.4	63%	NP_477067.2
sickie, isoform I (Drosophila melanogaster)	26.5	299	68%	9.4	45%	NP_001260606.1
uncharacterized protein Dmel_CG14454, isoform A (Drosophila melanogaster)	26.1	92.9	28%	11	61%	NP_649404.1
uncharacterized protein Dmel_CG12522 (Drosophila melanogaster)	26.1	93.1	41%	11	47%	NP_648438.1
new glue 3 (Drosophila melanogaster)	26.1	133	28%	11	56%	NP_525060.1
grain, isoform A (Drosophila melanogaster)	26.1	149	72%	12	50%	NP_731211.1
runt, isoform B (Drosophila melanogaster)	26.1	73.0	40%	12	47%	NP_001245786.1
runt, isoform A (Drosophila melanogaster)	26.1	73.0	40%	12	47%	NP_523424.2
invected, isoform B (Drosophila melanogaster)	26.1	96.7	66%	12	35%	NP_523699.3
grain, isoform B (Drosophila melanogaster)	26.1	249	71%	12	50%	NP_001138026.1
uncharacterized protein Dmel_CG9518, isoform A (Drosophila melanogaster)	26.1	56.6	67%	12	36%	NP_572979.1
skeletor, isoform D (Drosophila melanogaster)	26.1	26.1	21%	12	65%	NP_001027173.2
grain, isoform C (Drosophila melanogaster)	26.1	249	71%	12	50%	NP_001262366.1
uncharacterized protein Dmel_CG15473 (Drosophila melanogaster)	26.1	90.6	38%	12	45%	NP_572179.1
uncharacterized protein Dmel_CG15322, isoform B (Drosophila melanogaster)	26.1	161	33%	12	74%	NP_608371.2
protein tyrosine phosphatase 99A, isoform G (Drosophila melanogaster)	26.1	53.2	31%	13	50%	NP_001189309.1
scribbler, isoform C (Drosophila melanogaster)	26.1	318	55%	13	45%	NP_995884.1
scribbler, isoform F (Drosophila melanogaster)	26.1	334	55%	13	45%	NP_995883.1
ubiquitin protein ligase E3A (Drosophila melanogaster)	26.1	90.6	51%	13	52%	NP_648452.1
pou domain motif 3, isoform A (Drosophila melanogaster)	26.1	159	38%	13	43%	NP_610377.1
pico, isoform B (Drosophila melanogaster)	26.1	162	40%	13	59%	NP_728307.1
mediator complex subunit 1, isoform B (Drosophila melanogaster)	26.1	235	55%	13	41%	NP_001189153.1
pico, isoform C (Drosophila melanogaster)	26.1	205	52%	13	59%	NP_001188674.1
uncharacterized protein Dmel_CG8176, isoform A (Drosophila melanogaster)	26.1	189	55%	13	50%	NP_788612.1
mediator complex subunit 1, isoform C (Drosophila melanogaster)	26.1	270	55%	13	41%	NP_001262183.1
uncharacterized protein Dmel_CG8176, isoform G (Drosophila melanogaster)	26.1	142	48%	13	50%	NP_001262419.1
pico, isoform A (Drosophila melanogaster)	26.1	205	52%	13	59%	NP_608363.1
uncharacterized protein Dmel_CG8176, isoform C (Drosophila melanogaster)	26.1	189	55%	13	50%	NP_001097723.1

uncharacterized protein Dmel_CG4835 (Drosophila melanogaster)	26.1	462	48%	13	48%	NP_647965.3
protein tyrosine phosphatase 99A, isoform A (Drosophila melanogaster)	26.1	76.8	50%	13	50%	NP_733288.1
protein tyrosine phosphatase 99A, isoform C (Drosophila melanogaster)	26.1	76.8	50%	13	50%	NP_996302.1
thin, isoform D (Drosophila melanogaster)	26.1	301	52%	13	63%	NP_001286596.1
pou domain motif 3, isoform E (Drosophila melanogaster)	26.1	290	43%	13	43%	NP_001286193.1
thin, isoform E (Drosophila melanogaster)	26.1	321	52%	13	63%	NP_001286597.1
pou domain motif 3, isoform C (Drosophila melanogaster)	26.1	290	43%	13	43%	NP_001137617.2
pou domain motif 3, isoform F (Drosophila melanogaster)	26.1	290	43%	13	43%	NP_001188885.2
protein tyrosine phosphatase 99A, isoform B (Drosophila melanogaster)	26.1	90.1	50%	13	50%	NP_476816.1
thin, isoform A (Drosophila melanogaster)	26.1	301	52%	13	63%	NP_611390.2
protein tyrosine phosphatase 99A, isoform F (Drosophila melanogaster)	26.1	58.3	47%	13	50%	NP_651691.4
mediator complex subunit 1, isoform A (Drosophila melanogaster)	26.1	401	75%	13	41%	NP_649341.1
skeletor, isoform E (Drosophila melanogaster)	26.1	42.0	27%	13	65%	NP_001247027.1
thin, isoform C (Drosophila melanogaster)	26.1	316	52%	13	63%	NP_001137707.1
protein 1 of cleavage and polyadenylation factor 1, isoform E (Drosophila melanogaster)	26.1	235	43%	13	43%	NP_001188931.1
protein 1 of cleavage and polyadenylation factor 1, isoform F (Drosophila melanogaster)	26.1	236	46%	13	43%	NP_001286428.1
protein 1 of cleavage and polyadenylation factor 1, isoform G (Drosophila melanogaster)	26.1	235	43%	13	43%	NP_001286429.1
protein 1 of cleavage and polyadenylation factor 1, isoform D (Drosophila melanogaster)	26.1	235	43%	13	43%	NP_610999.4
uncharacterized protein Dmel_CG9932, isoform E (Drosophila melanogaster)	26.1	328	48%	13	61%	NP_001260429.1
uncharacterized protein Dmel_CG9932, isoform A (Drosophila melanogaster)	26.1	328	48%	13	61%	NP_609599.1
scribbler, isoform D (Drosophila melanogaster)	26.1	382	60%	13	45%	NP_001163189.1
scribbler, isoform G (Drosophila melanogaster)	26.1	397	60%	13	45%	NP_001163191.1
scribbler, isoform J (Drosophila melanogaster)	26.1	397	60%	13	45%	NP_001261074.1
uncharacterized protein Dmel_CG7656, isoform A (Drosophila melanogaster)	25.7	45.0	32%	16	52%	NP_730059.1
uncharacterized protein Dmel_CG7656, isoform D (Drosophila melanogaster)	25.7	45.0	32%	16	52%	NP_730058.2
pangolin, isoform Q (Drosophila melanogaster)	25.7	25.7	20%	17	63%	NP_001245405.1
mucin 4B, isoform B (Drosophila melanogaster)	25.7	355	65%	17	63%	NP_726915.2
RNA-binding protein 6, isoform B (Drosophila melanogaster)	25.7	116	46%	17	57%	NP_001097631.1

Sp1, isoform D (Drosophila melanogaster)	25.7	151	38%	17	57%	NP_001096927.2
Sp1, isoform E (Drosophila melanogaster)	25.7	151	38%	17	57%	NP_572579.3
Sp1, isoform F (Drosophila melanogaster)	25.7	151	38%	17	57%	NP_001259388.1
uncharacterized protein Dmel_CG6424, isoform A (Drosophila melanogaster)	25.7	90.6	45%	17	61%	NP_725709.1
uncharacterized protein Dmel_CG13643, isoform E (Drosophila melanogaster)	25.7	114	30%	17	62%	NP_001262938.1
uncharacterized protein Dmel_CG13643, isoform B (Drosophila melanogaster)	25.7	114	30%	17	62%	NP_652253.1
uncharacterized protein Dmel_CG13643, isoform D (Drosophila melanogaster)	25.7	114	30%	17	62%	NP_001262937.1
kin of irre, isoform A (Drosophila melanogaster)	25.7	89.3	57%	17	50%	NP_726844.1
uncharacterized protein Dmel_CG43736, isoform G (Drosophila melanogaster)	25.7	120	41%	17	41%	NP_001259297.1
uncharacterized protein Dmel_CG43736, isoform F (Drosophila melanogaster)	25.7	170	57%	17	41%	NP_001259296.1
tripeptidyl-peptidase II, isoform A (Drosophila melanogaster)	25.7	95.5	57%	17	52%	NP_477247.1
tripeptidyl-peptidase II, isoform E (Drosophila melanogaster)	25.7	95.5	57%	17	52%	NP_001286377.1
tripeptidyl-peptidase II, isoform D (Drosophila melanogaster)	25.7	95.5	57%	17	52%	NP_725252.1
pacman, isoform A (Drosophila melanogaster)	25.7	75.9	38%	17	71%	NP_523408.2
pacman, isoform B (Drosophila melanogaster)	25.7	75.9	38%	17	71%	NP_001162796.1
Myocardin-related transcription factor, isoform H (Drosophila melanogaster)	25.7	163	91%	17	55%	NP_001137879.3
hangover, isoform A (Drosophila melanogaster)	25.7	196	40%	17	90%	NP_727980.2
hangover, isoform D (Drosophila melanogaster)	25.7	214	45%	17	90%	NP_727979.3
hangover, isoform C (Drosophila melanogaster)	25.7	281	71%	17	90%	NP_001138204.1
toutatis, isoform A (Drosophila melanogaster)	25.7	314	77%	17	52%	NP_523701.3
toutatis, isoform F (Drosophila melanogaster)	25.7	314	77%	17	52%	NP_001260898.1
toutatis, isoform G (Drosophila melanogaster)	25.7	314	77%	17	52%	NP_001260899.1
toutatis, isoform E (Drosophila melanogaster)	25.7	314	77%	17	52%	NP_001097270.1
uncharacterized protein Dmel_CG11122 (Drosophila melanogaster)	25.7	343	52%	17	65%	NP_572685.2
huntingtin, isoform A (Drosophila melanogaster)	25.7	276	63%	17	45%	NP_651629.1
dumpy, isoform J (Drosophila melanogaster)	25.7	1466	57%	17	50%	NP_001245876.1
dumpy, isoform I (Drosophila melanogaster)	25.7	1466	57%	17	50%	NP_001245875.1
dumpy, isoform S (Drosophila melanogaster)	25.7	252	55%	17	50%	NP_001260034.1

dumpy, isoform AA (Drosophila melanogaster)	25.7	1523	57%	17	50%	NP_001260042.1
dumpy, isoform V (Drosophila melanogaster)	25.7	1509	45%	17	50%	NP_001260037.1
dumpy, isoform P (Drosophila melanogaster)	25.7	1538	57%	17	50%	NP_001260031.1
dumpy, isoform U (Drosophila melanogaster)	25.7	1552	57%	17	50%	NP_001260036.1
dumpy, isoform T (Drosophila melanogaster)	25.7	1552	57%	17	50%	NP_001260035.1
dumpy, isoform R (Drosophila melanogaster)	25.7	1552	57%	17	50%	NP_001260033.1
dumpy, isoform Q (Drosophila melanogaster)	25.7	1552	57%	17	50%	NP_001260032.1
uncharacterized protein Dmel_CG13947 (Drosophila melanogaster)	25.2	67.2	23%	20	64%	NP_608542.1
uncharacterized protein Dmel_CG42525, isoform B (Drosophila melanogaster)	25.2	247	57%	22	49%	NP_001261361.1
uncharacterized protein Dmel_CG42525, isoform A (Drosophila melanogaster)	25.2	282	67%	22	37%	NP_001163335.1
ELL-associated factor, isoform B (Drosophila melanogaster)	25.2	85.5	75%	22	36%	NP_724550.1

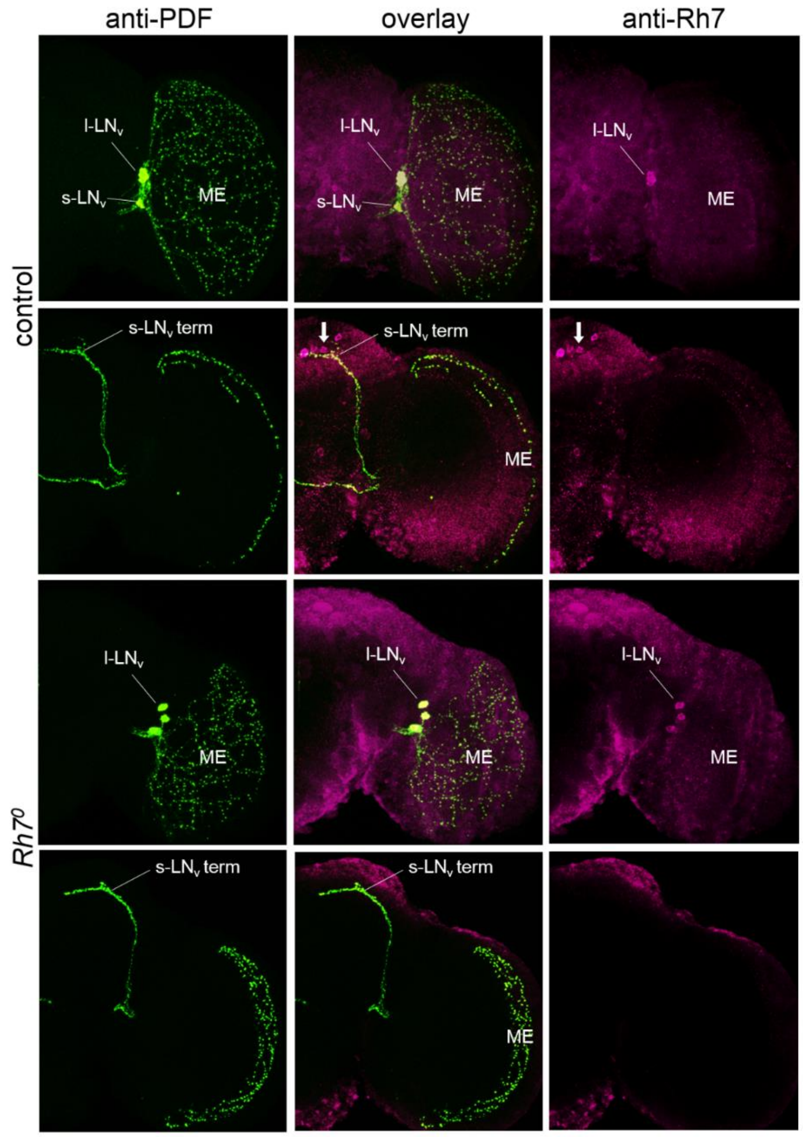

Figure S1. PDF and Rh7 labelling in the right brain hemispheres of a control fly and an *Rh7⁰* mutant. For each fly, the upper row shows the overlay of thirty anterior confocal sections (2µm thick) and the lower row shows thirty posterior sections. Rh7-labeling is present in the large PDF neurons ($l\text{-}LN_v$) of the control and the *Rh7⁰* fly. In the control fly, Rh7-labeling is additionally present in the neurons (white arrows), in the dorsal brain close to the terminals of the small PDF neurons ($s\text{-}LN_v$ term). ME medulla.
